# Restoration of normal embryogenesis by mitochondrial supplementation in pig oocytes exhibiting mitochondrial DNA deficiency

**DOI:** 10.1038/srep23229

**Published:** 2016-03-18

**Authors:** Gael L. M. Cagnone, Te-Sha Tsai, Yogeshwar Makanji, Pamela Matthews, Jodee Gould, Michael S. Bonkowski, Kirstin D. Elgass, Ashley S. A. Wong, Lindsay E. Wu, Matthew McKenzie, David A. Sinclair, Justin C. St. John

**Affiliations:** 1 Centre for Genetic Diseases, Hudson Institute of Medical Research, 27-31 Wright Street, Clayton, Vic 3168, Australia; 2 Department of Molecular and Translational Science, Monash University, 27-31 Wright Street, Clayton, Vic 3168, Australia; 3 Centre for Innate Immunity and Infectious Diseases, Hudson Institute of Medical Research, 27-31 Wright Street, Clayton, Vic 3168, Australia; 4 Department of Genetics, Harvard Medical School, 77 Avenue Louis Pasteur, Boston, MA, 02115, USA; 5 Monash Micro Imaging, Monash University, 27-31 Wright Street, Vic 3168 Clayton, Australia; 6 School of Medical Sciences, University of New South Wales, NSW 2052, Australia

## Abstract

An increasing number of women fail to achieve pregnancy due to either failed fertilization or embryo arrest during preimplantation development. This often results from decreased oocyte quality. Indeed, reduced mitochondrial DNA copy number (mitochondrial DNA deficiency) may disrupt oocyte quality in some women. To overcome mitochondrial DNA deficiency, whilst maintaining genetic identity, we supplemented pig oocytes selected for mitochondrial DNA deficiency, reduced cytoplasmic maturation and lower developmental competence, with autologous populations of mitochondrial isolate at fertilization. Supplementation increased development to blastocyst, the final stage of preimplantation development, and promoted mitochondrial DNA replication prior to embryonic genome activation in mitochondrial DNA deficient oocytes but not in oocytes with normal levels of mitochondrial DNA. Blastocysts exhibited transcriptome profiles more closely resembling those of blastocysts from developmentally competent oocytes. Furthermore, mitochondrial supplementation reduced gene expression patterns associated with metabolic disorders that were identified in blastocysts from mitochondrial DNA deficient oocytes. These results demonstrate the importance of the oocyte’s mitochondrial DNA investment in fertilization outcome and subsequent embryo development to mitochondrial DNA deficient oocytes.

Mitochondrial DNA (mtDNA) is a double-stranded circular genome that is approximately 16.6 kb in size and is located in the mitochondrial matrix^1^. It encodes 13 of the 80+ subunits of the electron transfer chain (ETC), which generates the vast majority of cellular ATP through oxidative phosphorylation (OXPHOS)^2^,^3^. The remaining OXPHOS subunits are encoded by the chromosomal genome. The mitochondrial genome also encodes 22 tRNAs and 2 rRNAs and has one non-coding region, the D-Loop, which is the site of interaction for the nuclear-encoded transcription and replication factors that translocate to the mitochondrion to first drive mtDNA transcription then replication^4^. Cells possess multiple copies of mtDNA, which are inherited from the population present in the oocyte at fertilization and passed from generation to generation through the female germline^5^.

There are a number of mtDNA disorders^6^, which include mtDNA deficiency syndromes that manifest in somatic tissues and organs and primarily affect cells that are highly dependent on OXPHOS for the generation of ATP^6^. Maturing mammalian oocytes and developing embryos are not highly dependent on OXPHOS. Their mitochondria are structurally and functionally quiescent, and they likely derive most of their energy through alternative pathways, such as the adenosine salvage pathway^7^. They are also involved in a number of cellular functions including the sequestration and release of intracellular calcium. Furthermore, women harboring severe mtDNA mutations retain the capacity to be fertile^8^ hence the persistence of mild and severe forms of mtDNA disease^6^.

Whilst experimental reduction of mtDNA copy number does not impair preimplantation embryo development in mice^9^, mtDNA depletion during pig oocyte *in vitro* maturation results in fertilisation failure or arrest during preimplantation development^10^. In addition, mtDNA deficiency appears to affect maturing pig oocytes resulting in their failure to complete nuclear and cytoplasmic maturation, which renders them developmentally incompetent^10^,^11^. Furthermore, human oocytes with low mtDNA copy number frequently fail to fertilise or arrest during preimplantation development^12^,^13^,^14^,^15^,^16^,^17^. To this extent, the amount of mtDNA present in the oocyte at fertilisation is likely to be an investment in subsequent developmental events. For example, during pig oocyte maturation, replication of mtDNA establishes a minimum investment of ~120 000 copies in oocytes that have the capacity to be fertilised^10^,^11^,^18^,^19^,^20^. This investment ensures that sufficient mtDNA is available during organogenesis so that each mature cell type has sufficient mtDNA copy number to meet its required metabolic demands, in a cell specific manner. This investment is important, as there is one brief mtDNA replication event that occurs between fertilisation and the 2-cell stage. However, mtDNA replication does not then occur during preimplantation development until the blastocyst stage, and then not again in embryonic cells until post-gastrulation^10^,^18^,^19^,^20^,^21^.

Supplementing oocytes with mitochondria is a strategy to overcome mtDNA deficiency and enhance developmental competence. Indeed, supplementation of mtDNA deficient oocytes with autologous populations of mitochondrial isolate can enhance fertilisation outcome, reinforcing the relationship between mtDNA copy number and oocyte development^11^. Moreover, autologous supplementation would prevent the transmission of two populations of mtDNA, known as heteroplasmy, that arose following the transfer of donor cytoplasm into oocytes of women with repeated embryonic development failure^22^ and led to the associated developmental disorders that impact on offspring health and survival^23^,^24^.

Oocytes can be selected by staining with Brilliant Cresyl Blue (BCB), a non-toxic dye that is reduced to a colorless compound by glucose-6-phosphate dehydrogenase (G6PD)^25^. As G6PD shows progressive down-regulation during oocyte growth, developmentally competent oocytes stain blue (BCB^+^ ) whilst developmentally incompetent oocytes are colorless (BCB^−^)^25^. To this extent, BCB staining has been used in various mammalian species to assess developmental competence^25^,^26^. In addition, pig BCB^+^ oocytes contain significantly higher levels of mtDNA copy number than BCB^−^ oocytes, as part of their differential competence to fertilise^10^,^11^. Indeed, the pig is an excellent model of oocyte and embryo development as these are very similar to that of the human^27^,^28^. In addition, mtDNA replication and reduction events have been mapped in porcine oocytes and embryos^10^,^11^, which are very similar to human oocytes and embryos^10^,^11^,^14^.

By supplementing BCB^−^ oocytes with autologous populations of mitochondrial isolate, we can rescue a significant proportion of oocytes, which enables them to progress to blastocyst. The impact is immediate with 2-cell stage embryos possessing enhanced levels of mtDNA. By enhancing BCB^−^ oocytes with mtDNA, gene expression patterns in blastocysts are more similar to embryos originating from developmentally competent rather than developmentally incompetent oocytes.

## Results

### Nuclear and cytoplasmic maturation in BCB^+^ and BCB^−^ oocytes

In an ovary, typically 38.7% ± 2.1 (mean ± SEM) oocytes stain negatively for BCB (BCB^−^). To validate the use of BCB staining as a differential marker of oocyte maturation for oocytes that had not been synchronised to the S-phase of the cell cycle^10^ (Supplementary Fig. 1), aspirated BCB^+^ and BCB^−^ oocytes were assessed for Metaphase II (MII; polar body extrusion) after 44 hr of *in vitro* maturation (IVM). Significantly more BCB^+^ oocytes (51.4%) developed to MII than BCB^−^ oocytes (20.3%; P < 0.0001; Supplementary Table 1), whilst lysis was higher in the BCB^−^ population (51.2%; P < 0.0001) (Supplementary Table 1).

**Figure 1:**
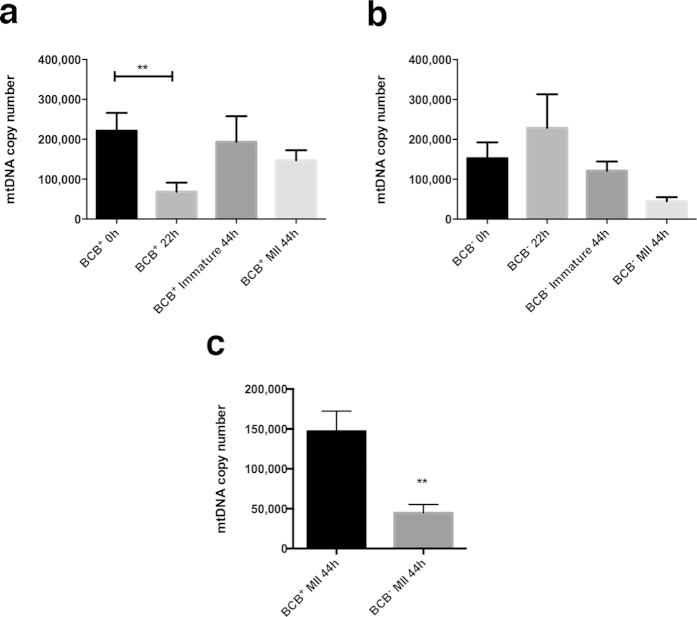
Mean (±SEM) mtDNA copy number in maturing and mature oocytes determined by real time PCR. (**a**) BCB^+^ and (**b**) BCB^−^ maturing (0 hr and 22 hr) and immature (44 hr) and mature (44 hr) oocytes (n = 10 for each). Statistical analysis was performed using ANOVA. (**c**) Comparison between BCB^+^ and BCB^−^ MII oocytes performed by t-test.

Real-time PCR was performed on individual *in vitro* matured BCB^+^ and BCB^−^ oocytes at 0, 22 and 44 hr post-isolation from ovaries. BCB^+^ oocytes had high copy number at 0 hr, which decreased significantly at 22 hr (P < 0.01) and returned to higher levels at 44 hr (Fig. 1a). This was the case for oocytes that reached and failed to reach MII. BCB^−^ oocytes behaved in the opposite manner with lower copy number at 0 hr followed by an increase at 22 hr and a return to far lower levels at 44 hr (Fig. 1b), demonstrating their incapacity to mediate nucleo-cytoplasmic interactions. The few BCB^−^ oocytes reaching MII had lower mtDNA copy number than immature oocytes at 44 hr. A direct comparison of MII BCB^+^ and BCB^−^ oocytes at 44 hr showed that BCB^−^ oocytes had significantly fewer copies of mtDNA (P < 0.01; Fig. 1c).

Furthermore, mitochondria isolated from BCB^+^ oocytes exhibited lower respiration rates than mitochondria from BCB^−^ oocytes when stimulated with succinate and ADP, but higher respiratory capacity when uncoupled with FCCP (Supplementary Fig. 2). BCB^+^ mitochondria were also in a more quiescent state than their BCB^−^ counterparts. In addition, there were distinct profiles for the distribution and clustering of mitochondria in BCB^+^ and BCB^−^ oocytes following staining with MitoTracker Deep Red (Supplementary Fig. 3). When cluster area, perimeter and circularity were chosen as parameters for characterization, there were significant differences between cluster area and perimeter for the two populations of oocytes.

### Supplementation of BCB^−^ oocytes with isolated mitochondria

**Figure 2:**
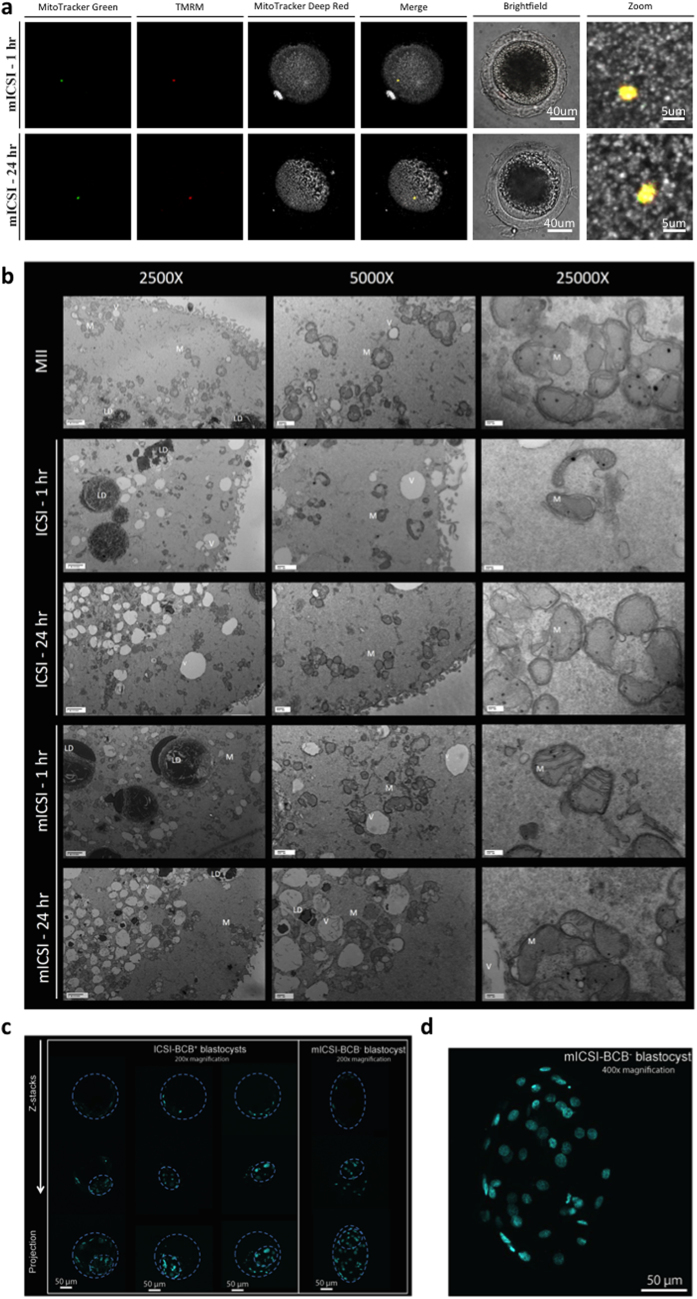
Generation of mICSI-derived blastocysts. (**a**) Localization of mitochondria following injection into BCB^−^ MII oocytes at 1 hr and 24 hr post insemination. Mitochondria endogenous to the oocyte are labeled with MitoTracker Deep Red (grey). The injected mitochondria are labeled with TMRM (red) and MitoTracker Green (green). The confocal z-stacks are displayed as maximum intensity projections (X40 magnification). (**b**) Transmission Electron Microscopy of MII oocytes, ICSI-1 hr, ICSI-24 hr, mICSI-1 hr, and mICSI-24 hr at 2500X, 5000X and 25,000X magnification. Abreviations include; LD = lipid droplet, V = vacuole, M = mitochondria. (**c**) DAPI staining of ICSI BCB^+^ and mICSI BCB^−^ blastocysts highlighting the presence of an inner cell mass. (**d**) Enlargement of a mICSI BCB^−^ blastocyst.

Using cohorts of MII BCB^+^ and BCB^−^ oocytes, we generated a series of blastocyst stage embryos through *in vitro* fertilisation (IVF), intracytoplasmic sperm injection (ICSI) and mitochondrial supplementation in combination with ICSI (mICSI) (Table 1). We generated a mitochondrial isolate from 5 to 10 BCB^+^ metaphase II oocytes for each ovary pair. The number of mtDNA copies delivered to each oocyte at 44 hr of IVM was 787.5 ± 409.3 with 44 hr deemed the most appropriate time as BCB^−^ oocytes showed large fluctuations in mtDNA copy number during the first 18 to 28 hr of IVM when compared to BCB^+^ oocytes (Supplementary Fig. 4). Indeed, supplemented mitochondria were tightly compact at 0 to 1 hr post-insemination and maintained their viability (Fig. 2a). At 24 hr, they were still present and viable within the cytoplasm and were beginning to exhibit less of a clustered feature (Fig. 2a). This was corroborated by transmission electron microscopy where quiescent mitochondrial morphology and dense organelles with translucent cristae were observed (Fig. 2b). We did find some mitochondria with more defined and possibly more biologically active cristae in the BCB^−^ mISCI group at 1 hr post injection, but these mitochondria where no longer detectable at 24 hr.

BCB^+^ oocytes inseminated by ICSI had the highest fertilisation rates (77.7%), as did BCB^−^ oocytes, compared with IVF (59.9% vs 38.1%; Table 1). Blastocyst rates were not significantly different following insemination by IVF, ICSI and mICSI for the BCB^+^ group. For the BCB^−^ group, mICSI led to significantly higher blastocyst rates (31.5%) compared with IVF (7.6%; P < 0.05) and improved blastocyst rates compared with ICSI (23.9%). Blastocyst rates for BCB^−^ oocytes inseminated by mICSI were very similar to BCB^+^ blastocysts generated by ICSI and mICSI. Furthermore, BCB^−^ mICSI-derived blastocysts had well-defined blastomeres with higher cell numbers (n = 51) than BCB^+^ ICSI-derived blastocysts (n = 33; Fig. 2c,d).

### MtDNA replication during preimplantation development

**Figure 3:**
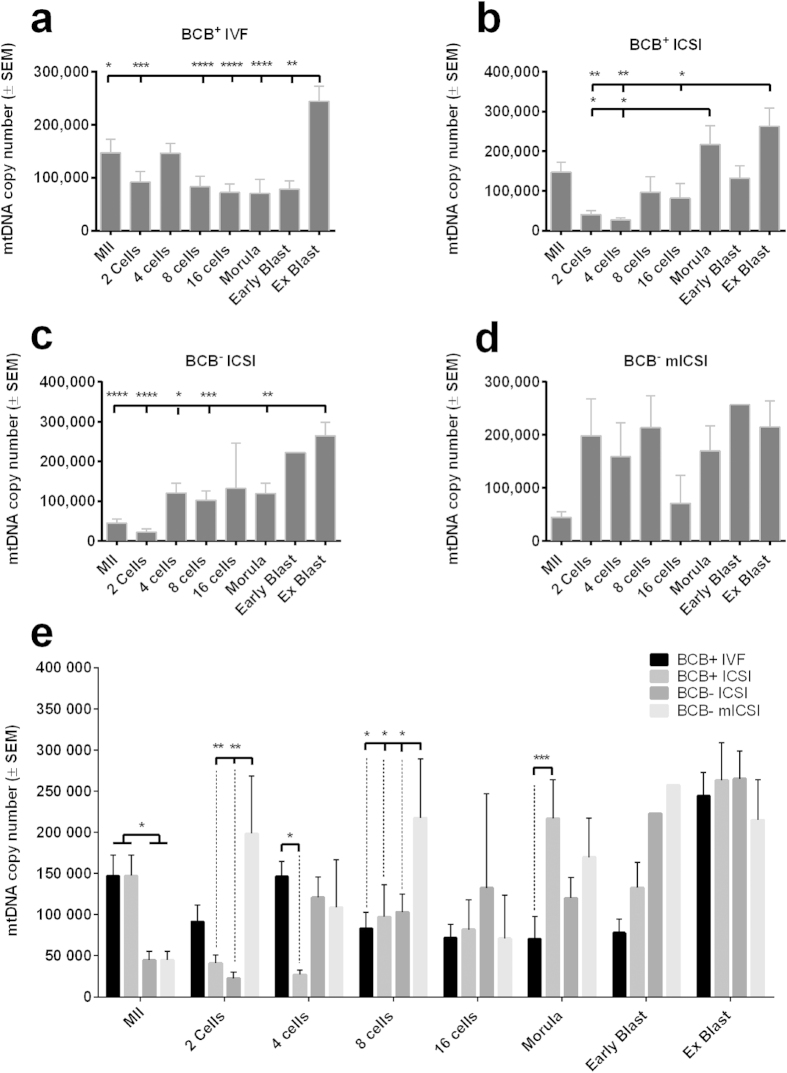
MtDNA copy number for preimplantation embryos. (**a**) Mean (±SEM) mtDNA copy number for BCB^+^ IVF; (**b**) BCB^+^ ICSI; (**c**) BCB^−^ ICSI and (**d**)BCB^−^ mICSI-derived embryos determined by real time PCR (n = 5–10). (**e**) Mean (±SEM) mtDNA copy for each stage of development for embryos generated by IVF, ICSI and mICSI from BCB^+^ and BCB^−^ oocytes.

To determine whether mtDNA was differentially replicated during preimplantation development following each treatment, copy number was assessed from the 2-cell to the blastocyst stage. Although there was a slight turnover at the 4-cell stage in BCB^+^ IVF generated embryos, copy number decreased during preimplantation development (Fig. 3a, P < 0.001) until a significant increase at the expanded blastocyst stage (Fig. 3a, P < 0.05). In BCB^+^ ICSI generated embryos, mtDNA copy number decreased at the 2-and 4-cell stages with significant expansion at the morula (Fig. 3b; P < 0.05) and expanded blastocyst stages (P < 0.01). For BCB^+^ oocytes, there was a significant turnover of mtDNA copy number at the 4-cell stage after IVF but not ICSI (Fig. 3e; P < 0.05). At later stages (Fig. 3e), there was significantly higher copy number in ICSI-generated compacting morulae than from IVF (P < 0.001). The profile for the ICSI-derived BCB^−^ embryos was very different to the IVF- and ICSI generated BCB^+^ embryos (*cf.* Fig. 3a–c,e).

BCB^−^ mICSI-generated embryos also exhibited very different profiles (Fig. 3d). Supplementation led to significantly higher mtDNA copy number at the 2-cell stage compared to ICSI (p < 0.01; Fig. 3d,e). This represented a 4.4-fold increase (Table 2), whilst ICSI-generated 2 cell embryos retained only 27.8% (BCB^+^ ) and 50.1% (BCB^−^) of their initial mtDNA copy number. Likewise, there were significant differences in mICSI 8-cell embryos compared to the other treatments (Fig. 3e). For day 7 expanded blastocysts, mtDNA copy number was > 200,000 copies following each treatment (Fig. 3e). For mICSI BCB^−^ blastocysts, this represented a 4.8-fold increase from the MII oocyte stage (Table 2) whilst for BCB^+^ blastocysts generated through IVF and ICSI, the increases were 1.7-fold and 1.8-fold, respectively.

### Global analysis of differential gene expression

**Figure 4:**
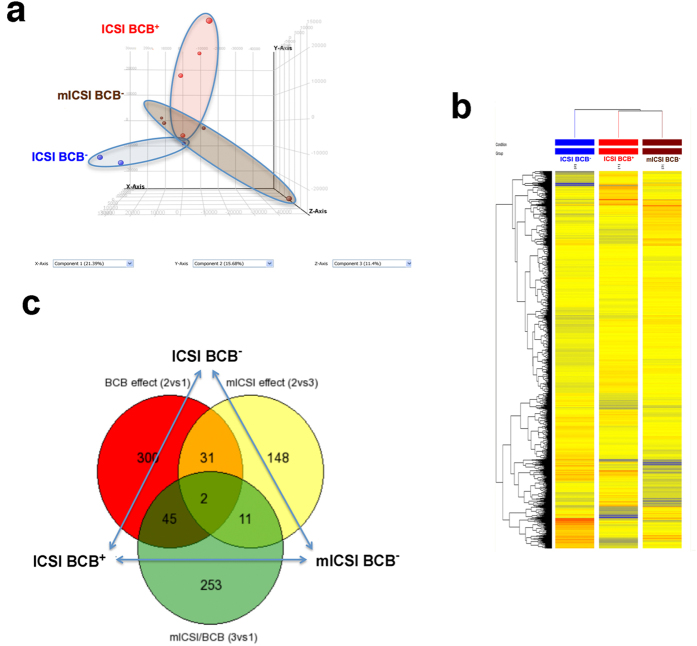
Global gene expression analysis of single blastocysts following microarray. (**a**) PCA of microarray data from single blastocysts. Red, blue and brown points represent individual transcriptomes from ICSI BCB^+^ , ICSI BCB^−^ and mICSI BCB^−^ blastocysts, respectively. (**b**) Heat map of global gene expression following Pearson’s correlation to determine hierarchical clustering between blastocysts of each group. (**c**) Venn diagram representing differentially expressed genes between ICSI BCB^+^ and ICSI BCB^−^; mICSI BCB^−^ and ICSI BCB^−^; and mICSI BCB^−^ and ICSI BCB^+^ blastocysts following unpaired t-tests, with FC > 2 (abs) and significance of p < 0.01.

To determine if mitochondrial transplantation modulated gene expression patterns in BCB^−^-derived blastocysts, global gene expression analysis was performed on single blastocysts generated from ICSI-BCB^+^ (n = 4), ICSI-BCB^−^ (n = 3) and mICSI-BCB^−^ (n = 4) oocytes. Using a linear RNA amplification protocol (Supplementary Fig. 5) and a global genome microarray, we assessed the expression of 28,944 probes across the different groups (Quantile normalisation, 20–100^th^ percentile and detection flag filters). Principal component analysis (PCA) demonstrated the clustering of replicates to their respective groups, except for one mICSI-BCB^−^ blastocyst outlier (Fig. 4a). Heat maps for gene expression also showed good clustering and greater similarity between the mICSI BCB^−^ and ICSI BCB^+^ blastocysts than the ICSI BCB^−^ blastocysts (Fig. 4b). Indeed, the probe intensities examined for genes associated with blastocyst development showed consistent expression of genes involved in pluripotency (Supplementary Fig. 6a), epigenetic reprogramming (Supplementary Fig. 6b), energy metabolism (Supplementary Fig. 6c,d) and microRNAs (Supplementary Fig. 6e) for mICSI BCB^−^ and ICSI BCB^+^ blastocysts.

Amongst each group of blastocysts, direct unpaired t-test comparisons identified several differentially expressed genes (DEG) (False Discovery Rate (FDR) = 0.05, Supplementary Table 2). There were 5 DEGs between BCB^−^ ICSI and BCB^+^ ICSI blastocysts, 7 DEGs between BCB^−^ mICSI and BCB^+^ ICSI blastocysts, and 1 DEG between BCB^−^ mICSI and BCB^−^ ICSI blastocysts. Across the three groups of blastocysts, ANOVA determined significant differences for 6 genes (FDR = 0.05, Supplementary Table 3). One DEG (predicted Ras and Rab interactor 3, LOC100155709) showed a greater fold change for the mICSI-BCB^−^ group than for the ICSI-BCB^−^ group compared to ICSI-BCB^+^ blastocysts. However, the other 5 DEGs (the putative olfactory receptor 51H1-like LOC100511338, the YKT6 v-SNARE homolog LOC100513964, the uncharacterized LOC100621956 and 2 novel genes) had greater fold change for ICSI-BCB^−^ than mICSI BCB^−^ blastocysts compared to ICSI-BCB^+^ blastocysts.

Analysis by ANOVA without FDR (p < 0.01, absolute fold-change ((FC)) > 2) produced a total of 309 DEGs with significant DEGs profiled according to relative fold change between the groups. This profiling showed greater fold-change differences in the ICSI-BCB^−^ blastocysts compared to ICSI-BCB^+^ or mICSI-BCB^−^ blastocysts (Supplementary Fig. 7a). In particular, ICSI-BCB^−^ blastocysts had a greater number of upregulated DEGs with fold changes higher than 5 or 10 (Supplementary Fig. 7b), whilst mICSI-BCB^−^ blastocysts showed a greater number of down-regulated DEGs (Supplementary Fig. 7c).

As large numbers of DEGs are required to reach sufficient enrichment for *in silico* pathway analysis, we compared pairs of groups without FDR but with FC > 2 and significance of p < 0.01 (Fig. 4c; and Table 3). Using Ingenuity Pathway Analysis, 276/378 DEGs were annotated for the comparison between ICSI BCB^−^ vs ICSI BCB^+^ blastocysts, which clustered into 12 networks (Supplementary Table 4). The top three networks were cellular assembly and organisation; cell morphology; and amino acid metabolism. For the cellular assembly and organisation pathway, a large number of genes were upregulated in the ICSI BCB^−^ blastocysts (Supplementary Fig. 8). Likewise, for the cell morphology (Supplementary Fig. 9) and the amino acid metabolism networks (Supplementary Fig. 10), a higher proportion of genes were upregulated in the ICSI BCB^−^ blastocysts than were down regulated. For the canonical pathways, PPAR signaling was the most affected (Supplementary Fig. 11). Of the predicted upstream regulators of the DEGs, CREB1, ERB2 and BMP2 were activated, as was the pathway that is modulated by the anti-Type 2 Diabetes pharmaceutical agent Troglitazone (Table 4; Supplementary Table 5).

In contrast, 127/192 DEGs were annotated from the comparison between mICSI BCB^−^ and ICSI BCB^−^ blastocysts, which clustered into seven networks (Supplementary Table 6). The cellular movement (Supplementary Fig. 12), cellular development (Supplementary Fig. 13) and cell morphology networks (Supplementary Fig. 14) ranked highest. The majority of genes in these networks were downregulated in the mICSI BCB^−^ cohorts. The PPAR canonical pathway was also affected in ICSI BCB^−^ blastocysts (Supplementary Fig. 15). Likewise, three predicted upstream regulators of the DEGs, namely NFKB, ILS and HRAS were significantly inhibited whilst the pathway modulated by the pharmaceutical agent resveratrol, which regulates Sirtuin activity and thus mitochondrial biogenesis 29, was activated (Table 4; Supplementary Table 7).

The comparison of mICSI BCB^−^ and ICSI BCB^+^ blastocysts resulted in 222/311 DEGs being annotated, which clustered into 10 networks (Supplementary Table 8). The highest ranked of these were cell cycle, cellular compromise and developmental disorders. For these networks, there was an improved balance between upregulated and downregulated DEGs compared to ICSI BCB^−^ blastocysts and more DEGs showed no marked differences. Of the canonical pathways, the regulators of metabolism were most affected (Table 4; Supplementary Fig. 16) whilst MYC and STAT4 were predicted upstream regulators to be activated (Supplementary Table 9), as was the pathway modulating the anti-cancer pharmaceutical agent Streptozocin.

We analysed genes that were not differently expressed between ICSI-BCB^+^ and mICSI-BCB^−^ but were differently expressed in ICSI-BCB^−^ blastocysts. 90/168 annotated genes clustered into 5 networks (Supplementary Table 10). The top three networks were cell morphology (Supplementary Fig. 17), gene expression and protein synthesis (Supplementary Fig. 18), and cell to cell signaling and interaction and cellular assembly and association (Supplementary Fig. 19). Glycine biosynthesis I, methylmalonyl pathway, and 2-oxobutanoate degradation I were the top three affected canonical pathways (Supplementary Fig. 20). However, no upstream regulators were significantly activated or inhibited (Supplementary Table 11).

### Analysis of patterns of gene expression specific to embryonic development

Cohorts of blastocysts (n = 5 to 10) were also analysed to determine the patterns of expression for key regulatory genes. Each of the pluripotent genes, OCT4 (Supplementary Fig. 21a), SOX2 (Supplementary Fig. 21b) and REX1 (Supplementary Fig. 21c) showed consistent levels of expression across the different groups, whilst NANOG expression was very low (Ct > 40) with no detection in mICSI BCB^+^ blastocysts (Supplementary Fig. 21d), as expected for porcine embryos^30^. The trophectodermal marker, CDX2, was consistently expressed in IVF, ICSI and mICSI blastocysts (Supplementary Fig. 21e). Two mtDNA-encoded genes, ND1 (Supplementary Fig. 21f) and ATP6 (Supplementary Fig. 21g), and the nuclear-encoded mtDNA transcription factor TFAM (Supplementary Fig. 21h) were highly expressed and within similar range.

## Discussion

The number of mtDNA copies present in developmentally competent MII oocytes is an essential investment in development, as embryonic cells only initiate mtDNA replication post-gastrulation^20^, a large number of cell divisions post-fertilisation. These are important developmental events as they establish the mtDNA set point from which all naïve cells possess low numbers of mtDNA^31^,^32^,^33^,^34^. These copies of mtDNA are then replicated in a cell specific manner in order that mature, specialised cells acquire their specified numbers of mtDNA copy to produce sufficient ATP through OXPHOS to perform their specialised functions. Indeed, homozygous knockdown mice that are unable to initiate mtDNA replication post-gastrulation die *in utero*^35^,^36^.

Although ~800 copies of mtDNA were injected into mtDNA deficient oocytes, MII oocytes are receptive to modulating mtDNA copy number to overcome their deficiency and promote development. MtDNA deficient oocytes ‘hitchhike’ on an embryo’s potential to replicate mtDNA prior to the 4-cell stage, which marks embryonic genome activation (EGA)^37^. This 4.4-fold increase at the 2-cell stage following mitochondrial supplementation stabilises mtDNA copy number for post-EGA development and ensures that numbers are similar to IVF- and ICSI- derived BCB^+^ blastocysts. Indeed, BCB^+^ oocytes did not benefit from supplementation suggesting that the appropriate threshold had been reached. Consequently, the replication events observed in IVF and ICSI generated BCB^+^ embryos ensure that sufficient mtDNA is present at the blastocyst stage and are essential to continued embryo development.

Previously, it has been demonstrated that the supplementation of oocytes with genetically distinct populations of mtDNA, through cytoplasmic transfer, results in heteroplasmy^22^,^23^. In mice, the health and wellbeing of the offspring was compromised by a host of physiological abnormalities^23^. Other studies in heteroplasmic mice have indicated similar outcomes^24^. The transfer of donor cytoplasm to treat women whose embryos undergo repetitive developmental arrest prior to or at EGA^38^,^39^,^40^,^41^ resulted in heteroplasmy at higher than anticipated levels even though the cytoplasmic extract was not a mitochondrial concentrate. Indeed, 40% of the offspring’s total mtDNA originated from the donor cytoplasm^22^,^42^, which supports our findings that mtDNA introduced at fertilisation ‘hitchhikes’ on the pre-EGA mtDNA replication event to provide sufficient mtDNA investment for subsequent development. Nevertheless, this approach led to autism and Turner’s syndrome (XO) but not in all cases^40^,^41^,^42^.

We propose that supplementation with autologous populations of mitochondrial isolate does not perturb the genetic identity of the offspring but generates a stabilisation effect, which is reflected in the enhanced gene expression patterns observed at the blastocyst stage. The gene expression patterns for BCB^−^ supplemented oocytes (mICSI BCB^−^) are far more aligned to the ICSI BCB^+^ cohort. The key gene networks positively affected in mICSI BCB^−^ blastocysts included cellular movement, cellular development and cell morphology, which are essential to developmental outcomes. These networks closely resemble deficits observed in ICSI BCB^−^ derived blastocysts that attempted to compensate by increasing gene expression associated with amino acid metabolism, which is essential to early development^27^.

The ‘quiet’ hypothesis suggests that early embryonic metabolism works at a quiet pace, but that insufficient metabolic support induces an adaptive response through increased gene expression that compromises embryonic development^43^. From the DEG profiles, it appears that BCB^−^ ICSI blastocysts had a higher number of upregulated genes compared to BCB^+^ ICSI blastocysts, with significant enrichment in the PPAR signaling pathway. This up-regulation is also predicted to involve activation of transcription factors, such as CREB1 (Table 4), an important metabolic regulator of blastocyst development^44^. As CREB1 is also involved in mitochondrial maintenance prior to EGA^45^ and energy homeostasis^46^, it would also communicate mtDNA deficiency to BCB^−^ embryos prior to EGA.

The upregulation of gene expression in BCB^−^ derived blastocysts was stabilised by mICSI and affected PPAR signalling, as well as other signalling pathways such as NKkB. Notably, mICSI down-regulated genes correlated with upstream activation by resveratrol (Table 4). Resveratrol is an exogenous compound beneficial to oocyte maturation and embryo development in mice^47^, cattle^48^ and pigs^29^. The effects of resveratrol involves activation of Sirt1, which influences mitochondrial biogenesis as well as mtDNA copy number in porcine oocytes^29^. Consequently, the effect of mICSI on blastocyst gene expression would be to communicate via retrograde signaling the stabilising effect of increased mtDNA copy number in early embryos. Collectively, these outcomes promote stabilised mtDNA replication under pluripotent gene control that is essential to the undifferentiated state, as observed in embryonic stem cells^31^,^49^,^50^. Likewise, the activation of MYC and STAT4 in mICSI BCB^−^ blastocysts, when compared to ICSI BCB^+^ blastocysts, is associated with increased cell proliferation in other cellular systems^51^, and, would account for the higher cell number observed in mICSI BCB^−^ blastocysts.

Whilst oocyte and early embryonic mitochondria do not fully mature before the blastocyst stage, we show here that the mtDNA content can be modulated throughout preimplantation development, and, particularly, prior to embryonic genome activation. In mtDNA deficient oocytes, this modulation does not occur which could account for lower blastocyst development. On the other hand, the increased mtDNA copy number in BCB^−^ oocytes following mICSI could counteract the homeostatic response to early mtDNA deficiency, and influence the embryonic development by stabilizing communication between the mitochondrial and nuclear genomes, which fails in other cellular systems, such as tumours, where the failure to efficiently replicate mtDNA prevents differentiation from taking place^52^. Whilst we argue that the increase in mtDNA copy number induces a stabilising effect on the embryo prior to embryonic genome activation, others have proposed that metabolic quiescence in oocytes and early embryos serves to protect the integrity of the mitochondrial genome^53^. If mitochondrial supplementation is to be introduced into clinical treatment, it would be pertinent to evaluate the potential for DNA damage by determining the levels of double strand breaks through, for example gamma H2AX labeling, or the comet assay, and to test for aneuploidy. This would demonstrate that there are no detrimental effects from supplementation on genome integrity and that, indeed, mtDNA supplementation is not inducing a metabolic affect that harms the embryo but, rather, supports subsequent development.

In conclusion, our outcomes demonstrate the importance of mtDNA to oocyte developmental competence, whereby supplementation of mtDNA deficient oocytes at fertilisation enhances embryo development and blastocyst quality. It also highlights the importance of inducing early mtDNA replication events at fertilisation in mtDNA deficient oocytes to enhance embryo quality by stabilising the embryo prior to EGA. This mtDNA investment ensures that resultant blastocysts have increased cell numbers and enhanced gene expression profiles that are associated with blastocysts from developmentally competent oocytes. We conclude that mitochondrial supplementation could significantly improve fertilization and development rates in mammals. In addition to advanced maternal age and poor ovarian reserve, this procedure could be relevant to the rescue of *in vitro* matured human oocytes^54^,^55^, notably in women with polycystic ovary syndrome^56^.

## Methods

All chemicals were obtained from Sigma, unless stated otherwise.

### BCB staining, IVM and embryo culture

Pig ovaries, excess to the requirements of the food chain, were collected from a local abattoir. They were washed and maintained in PBS at 37–38 °C. Cumulus-oocyte complexes (COCs) were aspirated from the ovaries using a syringe with an 18G needle (Becton Dickinson) containing around 1 ml warm flush medium (ViGRO, Bioniche Australia). After washing in pre-equilibrated IVM medium consisting of TCM 199, 0.1% polyvinyl alcohol, 3.05 mM Glucose, 0.91 mM Sodium Pyruvate, 0.57 mM Cysteine, 10 ng/mL EGF, 10 IU/mL LH (Chorulon, Intervet DE), 10 IU/mL FSH (Folligon, Intervet, DE) and 50 μg/mL Penicillin/streptomycin, COCs were stained with 12 μM BCB in IVM medium for 60 mins at 39 °C, 5% CO_2_ at maximum humidity. COCs were then washed in warm flush medium containing the supplements listed for IVM medium. BCB^+^ and BCB^−^ COCs were isolated using a stereomicroscope. After sorting, COCs were plated (50 per well) into pre-equilibrated IVM medium and incubated for 44 hours at 39 °C, 5% CO_2_ at maximum humidity.

After IVM, COCs underwent either IVF or ICSI followed by *in vitro* culture in Porcine Zygote Medium (PZM; saline solution containing 0.20 mM Sodium Pyruvate, 2 mM Calcium Lactate, 1 mM L-Glutamine, 5 mM Hypotaurine, Basal Medium Eagle and Non-essential amino-acids, 0.05 mg/ml penicillin/streptomycin and 0.3% BSA). For IVF, 50 COCs were plated in a 4-well plate containing pre-equilibrated mTBM solution (Tris Buffer Medium with 5 mM Sodium Pyruvate, 0.02 mM fresh Adenosine, 0.2 mM fresh L-Glutathione and 0.1% BSA) and incubated with 90% Percoll-pelleted spermatozoa (0.5 × 10^6^) for 4 hours at 39 °C, 5% CO_2_ at maximum humidity.

For ICSI, two injection plates were prepared with a central 7 μl drop of sperm catch (Nidacon SC-100), with a 1 μl arm of DPBS/FBS. This was surrounded by 4, 7 μl drops of pre-equilibrated PZM and overlayed with pre-equilibrated mineral oil (Sage 4008). Plates were equilibrated at 38.5 °C, 5% CO_2_, 5% O_2_ for at least 1 hour. Sperm was added to the PBS/FBS arm and allowed to swim into the sperm catch. Immediately prior to injection, a single oocyte was loaded into each of the PZM drops. ICSI was performed on a Nikon TE300 inverted microscope with a heated stage and fitted with Eppendorf micromanipulators. Injection and holding pipettes were supplied by The Pipette Company, Australia. Injection pipettes had an internal diameter of 6 μm. Individual sperm that had migrated into the sperm catch and were motile were immobilised by scoring the tail and drawn into the injection pipette. The injection pipette was moved to a drop containing an oocyte. The oocyte was held with the polar body orientated at either 6 or 12 o’ clock. The injection pipette with the sperm in the tip was introduced into the oocyte at 3 o’ clock and the cytoplasm aspirated to ensure the oolemma had been ruptured before depositing the sperm. Post-injected oocytes were transferred to pre-equilibrated PZM for culture at 38.5 °C, 5% CO_2_, 5% O_2_. mICSI was performed as for ICSI except that 3 pl of mitochondrial isolate was collected into the injection pipette along with a single sperm and injected at the time of sperm injection.

After insemination, zygotes were cultured in PZM for 7 days at 39 °C, 5% CO_2_, 5% O_2_ at maximum humidity with media changes after 48 and 120 hours. Imaging of DAPI-stained nuclei in expanded blastocysts was undertaken using a multi-photon confocal microscope (Leica TCS SP5).

### Mitochondrial isolation and supplementation

Mitochondrial isolation from *in vitro* matured metaphase II BCB^+^ oocytes was performed using a drill-fitted Teflon pestle^57^. Briefly, after hyaluronidase treatment, mechanical stripping and multiple PBS-washes to eliminate all cumulus cells, denuded oocytes were resuspended in 5 mL mitochondrial isolation buffer +2 mg/ml BSA (20 mM Hepes pH 7.6, 220 mM Mannitol, 70 mM sucrose, 1 mM EDTA) and homogenised by no more than 10 strokes of the pestle, at 4 °C or on ice. The oocyte homogenate was centrifuged at 800 g for 10 minutes to remove cell debris and the supernatant was centrifuged at 10,000 g for 20 minutes to pellet the mitochondrial fraction. The pellet was resuspended in isolation buffer without BSA then centrifuged at 10,000 g for 20 minutes. The supernatant was removed and the mitochondrial pellet resuspended in isolation buffer without BSA. The mitochondrial suspension was further concentrated by transferring it to a sealed straw (Flexipet, Cook Australia) followed by centrifugation for 25 seconds at 10,000 g. The mitochondrial suspension was dispensed onto an ICSI plate. At injection, a single sperm along with 3pl of mitochondrial isolate was drawn up into the injection pipette. The 3 pl of mitochondrial isolate along with the sperm was injected into the oocyte.

The mitochondrial isolate was quantified for mtDNA copy number by injecting an identical volume to that injected into oocytes into 2 μl of H_2_O within a PCR tube, and analysed in triplicate by real time PCR using mtDNA-specific primers, as described in ‘Analysis of mtDNA copy number’. Furthermore, the non-concentrated mitochondrial suspension (10 μl) underwent respiration analysis, as described in ‘Mitochondrial respiration analysis’.

### Mitochondrial respiration analysis

O_2_ consumption rates for isolated mitochondria were determined by high-resolution respirometry (Oroboros Oxygraph-2K, Innsbruck, Austria). The Oxygraph was calibrated at 0% (5% sodium hydrosulfide) and maximal (air) O_2_ concentration in mitochondrial respiration buffer (225 mM D-mannitol, 75 mM sucrose, 10 mM KCl, 10 mM Tris-HCl, 5 mM KH_2_PO_4_). After 2 minute washes of H_2_O, EtOH 80%, EtOH 100% and H_2_O respectively, the chambers were filled with 2 ml respiration buffer maintained at 37 °C and continuously stirred at 750 rpm. O_2_ consumption was measured using the integrated software package Datlab (Version 3.1; Oroboros, Innsbruck, Austria), which presented respiration as O_2_ flux, pmol O_2_ per unit per second. Following O_2_ flux stabilization, 25 μl of succinate (1 M) was added to the chamber as well as mitochondrial isolate and initial resting measurements were recorded. 10 μl of ADP (25 mM) followed by increasing doses of FCCP (carbonyl cyanide p-(trifluoromethoxy) phenylhydrazone) were added at 10 minute intervals to determine maximal uncoupled ETC respiratory capacity. Finally, 10 μl (5 mM) of the complex III inhibitor Antimycin A was used to abolish respiration.

### Analysis of mtDNA copy number

Individual denuded oocytes and embryos were freeze-thawed in 50 μl of water. 2 μl was added to 10 μl SYBR green (Bioline, Australia), 6 μl of H_2_O and 0.5 μM of each primer. See Supplementary Table 12 for primer sequences, product size, annealing temperature, and accession number. Reactions were run in a Rotorgene-3000 (Corbett Research, Cambridge, UK), according to the following conditions: 95 °C for 5 min, 45 cycles of melting temperature for 30 sec and acquisition temperature for 15 sec, followed by 1 cycle at ramping temperature from 72 °C to 95 °C with continuous fluorescence acquisition. All samples were analysed in triplicate. MtDNA copy number was extrapolated from standard curves (10-fold serial dilutions of 2 ng target PCR product) and calculated according to the target PCR product length. To determine the number of mtDNA copies introduced into oocytes by mICSI, the volume of mitochondrial isolate used to supplement the oocytes was directly added to a PCR tube and processed and analysed, as described in ‘Mitochondrial isolation and supplementation’.

### Imaging of mitochondria in inseminated oocytes

Denuded metaphase II oocytes were labeled with 100 nM MitoTracker Deep Red (Molecular Probes) in IVM medium for 45 min prior to injection. The mitochondrial isolate labeled with 200 nM MitoTracker Green FM (Molecular Probes) and 20 nM TMRM (Molecular Probes) was centrifuged at 10,000 g for 20 minutes. The mitochondrial pellet was washed to discard any unbound stain and concentrated as described in ‘Mitochondrial isolation and supplementation’. Injection was performed as described in ‘Mitochondrial isolation and supplementation’. Presumptive embryos were transferred into 8-well chamber slides on a coverslip (Sarstedt, product no. 94.6190.802).

Brightfield and MitoTracker Deep red images of presumptive embryos and fluorescent images of injected mitochondria were recorded on a Nikon C1 confocal microscope using a 40X oil immersion objective. 488 nm, 561 nm, and 639 nm lasers were used to specifically excite MitoTracker Green, TMRM and MitoTracker Deep Red in a sequential manner. Confocal z-stacks of 50 μm thickness with 5 μm increments between slices were recorded of the lower half of each oocyte.

### Shape Analysis of Mitochondrial Clusters

Oocytes were stained with 100 nM MitoTracker Deep Red (Molecular Probes) for 45 min and then transferred into 8-well chamber slides on a coverslip (Sarstedt, product no. 94.6190.802). Images of MitoTracker Deep Red were recorded on a Nikon C1 confocal microscope using a 40x oil immersion objective. Confocal z-stacks of 50 μm thickness with 5 μm increments between slices were recorded of the lower half of each oocyte.

Image processing and analysis was performed using Fiji software. For shape analysis of mitochondrial clusters, a suitable region of interest (ROI) with even staining of mitochondria and excluding any oocyte edges was chosen for each data set. The ROIs were then thresholded and binarized. Particle analysis was then performed to detect and characterize clusters. Cluster area, perimeter and circularity were chosen as parameters for characterization. Clusters at ROI edges were excluded from the analysis. A total of 7 BCB^−^ and 9 BCB^+^ oocytes and 402 and 651 mitochondrial clusters, respectively, were analyzed.

### Imaging of blastocysts to assess cell number

Day 7 blastocysts derived from ICSI BCB^+^ and mICSI BCB^−^ oocytes were fixed using 4% paraformaldehyde, then permeabilized in 1% TRITON X-100 and stained with DAPI. Image capture of DAPI stained blastocysts was performed by confocal microscopy using the multiphoton Leica SP8 (Leica Microsystems, Ontario Canada). Cell number represents the number of DAPI-stained nuclei per blastocyst.

### Transmission Electron Microscopy

Porcine eggs were fixed for at least 48 hours at MII, 1 hr and 24 hr post-ICSI and/or post-mICSI in freshly made 2% paraformaldehyde and 2.5% glutaraldehyde in 0.1 M sodium cacodylate buffer. Samples then underwent osmication and uranyl acetate staining, dehydration in alcohols and embedded in Taab 812 Resin (Marivac Ltd., Nova Scotia, Canada). Subsequent blocks were cut and sectioned with a Leica ultracut microtome, picked up on 100 mesh formvar/carbon coated Cu grids, stained with 0.2% lead citrate, and imaged under the Phillips Technai BioTwin Spirt electron microscope.

### RNA extraction, amplification and reverse transcription

RNA was extracted from single or pooled blastocysts using the Picopure RNA isolation Kit (Arcturus), according to the manufacturer’s instructions. Extracted RNA was treated with DNAse I to remove genomic DNA. RNA was amplified by 2 rounds of *in vitro* transcription (Arcturus RiboAmp HS PLUS, Life Technologies) or reverse-transcribed (random primers) using Superscript III (Life Technologies), according to the manufacturer’s instructions. After reverse-transcription, cDNA was diluted in 50 μl H_2_O and real time PCR was conducted using a Rotorgene-3000. Relative differences in Ct values were compared to IVF BCB^+^ blastocysts and normalized against ß-Actin (ACTB). Primer sequences, product size, annealing temperature, and accession numbers are provided in Supplementary Table 12.

### Microarray analysis

Highly diluted RNA Spike-Ins (Agilent #5188-5279, CA) were added at the time of RNA extraction to individual blastocysts^58^,^59^. Quality and concentration of extracted RNA were analyzed before and after T7 RNA amplification (Bio analyzer, Agilent). 1.8 μg amplified RNA was labeled with Cy3-ULS using the Agilent DNA ULS Fluorescent Labeling kit. The Degree of Labeling (DOL) was measured with the Nanodrop ND-1000 Spectrophotometer (Thermo Scientific). 1.65 μg of Cy3 labeled antisense RNA (aRNA; DOL 1–3.5%) was fragmented at 60 °C for 30 minutes in a reaction volume of 25 μl containing 1x Agilent fragmentation buffer and 4.4x Agilent blocking agent, according to the manufacturer’s instructions. On completion, 55 μl of 2x Agilent gene expression hybridisation buffer, 3 μl H_2_O and 27 μl CGH ULS block were added and 100 μl of the mixture was hybridised for 17 hours at 67 °C in a rotating Agilent hybridisation oven. After hybridisation, EmbryoGENE Porcine −4 X 44K (ID 031068) microarrays (Agilent Technologies, CA) were washed for 1 minute at room temperature with GE wash buffer 1 (Agilent) and 1 minute with 37 °C GE wash buffer 2 (Agilent, CA). Slides were scanned immediately after washing on the Agilent C DNA microarray scanner. The scanned images were analyzed using Feature Extraction Software 11.0.1.1 (Agilent). Ingenuity pathway analysis (IPA) was used to determine the signaling pathways underlying the transcriptomic differences (p < 0.05, abs. FC > 2) between blastocysts derived from ICSI BCB^+^ , ICSI BCB^−^ and mICSI BCB^−^ oocytes. Lists of DEGs from each direct comparison were prepared and imported into IPA by uploading lists of DEGs into separate experiments.

### Gene expression analysis in IPA

DEGs were matched with their universal gene symbols, then compiled into canonical pathways as well as gene product interactions (networks) that are developed from information contained in Ingenuity’s Knowledge Base. Canonical pathway analysis identified the pathways from the IPA library of canonical pathways that were most significant to the data set (enrichment score and probability). Networks of network-eligible molecules were algorithmically generated based on their connectivity. Green and red symbols represented genes respectively down-and up-regulated. DEGs were also submitted to upstream regulator analysis, which is based on prior knowledge of expected effects between transcriptional regulators and their target genes stored in the Ingenuity^®^ Knowledge Base. The analysis examines how many known targets of each transcription regulator are present in the dataset, and also compares their direction of change (i.e. expression in the experimental sample(s) relative to control) to what is expected from the literature in order to predict likely relevant transcriptional regulators. Each potential transcriptional regulator is given a p-value (probability to overlap with the data set) and an activation score (likelihood to be activated or inhibited with regard to the expression of target genes).

### Statistics

An unpaired t-test was used to compare mtDNA copy number between MII BCB^+^ and BCB^−^ oocytes and lysis rates. Direct comparison of global gene expression from microarray analysis was performed using an unpaired t-test corrected for false discovery rate (FDR) or filtered for absolute (abs.) fold-change >2 and p-value <0.01. One-way ANOVA was used to compare MII rates and lysis rates; blastocyst rates; mtDNA replication profiles during either IVM, preimplantation development or between BCB staining/treatments; and differential gene expression in BCB blastocysts generated by ICSI or mICSI. ANOVA results for differential gene expression were corrected for FDR. Using Agilent’s GeneSpring GX software, microarray data for each single blastocyst were subjected to principal component analysis and global gene expression was clustered by hierarchical Pearson’s correlation. Ingenuity Pathway Analysis (IPA, QIAGEN Redwood City, www.qiagen.com/ingenuity) was used to determine significant enrichment of biological pathways associated with differentially expressed genes. Statistical significance is represented as *, **, ***, **** for p values of <0.05, 0.01, 0.001, 0.0001, respectively.

## Additional Information

**Accession codes:** Data have been deposited into NCBI Gene Expression Omnibus (GEO): Gene expression microarrays (GSE65310).

**How to cite this article**: Cagnone, G. L. M. *et al.* Restoration of normal embryogenesis by mitochondrial supplementation in pig oocytes exhibiting mitochondrial DNA deficiency.. *Sci. Rep.* **6**, 23229; doi: 10.1038/srep23229 (2016).

## Supplementary Material

Supplementary Information

## Figures and Tables

**Table 1:** Development rates for BCB^+^ and BCB^−^ oocytes fertilised by IVF, ICSI and mICSI.

	Fertilisation	Total oocyte^*^ or MII number	% Fertilisation (total)	% Blastocyst/ Fert (total)	% Blastocyst/ Fert (±S.D)
BCB^+^	IVF	764^*^	58.4	23.7	20.6 ± 13.9
	ICSI	255	77.7	34.9	33 ± 15.3
	mICSI	98	62.2	31.6	31.5 ± 13.8
BCB^−^	IVF	507^*^	38.1	10.6	7.6 ± 5.8^a^
	ICSI	136	59.9	22	23.9 ± 11.0a,b
	mICSI	139	40.4	27.8	31.5 ± 15.6^b^

^a,b^Different superscripts represent significant differences, p < 0.05.

**Table 2:** MtDNA copy number in MII oocytes and 2-cell and blastocyst stage embryos.

Oocyte selection and fertilisation	mtDNA copy number ± SEM			Ratio	
	MII	2 cells	Blastocyst	2 cells/MII	Blastocyst/MII
BCB^+^ IVF	147063 ± 25325^a^	91630 ± 19950^a,b^	244245 ± 28765	62.3%	166.1%
BCB^+^ ICSI	147063 ± 25325^a^	40926 ± 10057^a^	263067 ± 45966	27.8%	178.9%
BCB^−^ ICSI	44749 ± 10574^b^	22419 ± 7588^a^	265000 ± 33329	50.1%	592.2%
BCB^+^ mICSI	147063 ± 25325^a^	nd	273712 ± 52787	nd	186.1%
BCB^−^ mICSI	44749 ± 10574^b^	198539 ± 69880^a^	214841 ± 49107	443.7%	480.1%

ANOVA between BCB^+^ and BCB^−^ for IVF, ICSI and mICSI inseminations with different superscripts representing significant differences, p < 0.05.

**Table 3:** Top 10 differentially expressed genes between ICSI BCB^+^ and ICSI BCB^−^; mICSI BCB^−^ and ICSI BCB^−^; and mICSI BCB^−^ and ICSI BCB^+^ blastocysts following unpaired t-tests, with FC > 2 (abs) and significance of p < 0.01.

Top 10 differentially expressed genes (T-Test, p value < 0.01, abs. FC > 2)	FC (abs)	p value	Regulation	REFSEQ	Network	Top Diseases and Functions
*ICSI BCB*^−^ *vs ICSI BCB*^*+*^*blastocysts*						
LOC100513964	109.53825	0.000014519855	up	XM_003134882	Not displayed	Undetermined
BNP	45.177853	0.005953322	up	NM_213846	Not displayed	Undetermined
NULL	34.279636	0.0000691877	up	unknown	Not displayed	Undetermined
ROCK2	29.54274	0.0013772815	up	XM_003125386	Supp. Fig. 8 and 18	Cell Morphology, Cellular Assembly and Organization, Cellular Function and Maintenance
LOC100518872	25.287235	0.0013035844	up	XM_003133613	Not displayed	Undetermined
NULL	77.53339	0.008828815	down	unknown	Not displayed	Undetermined
ZNF709	33.383026	0.0010354982	down	XM_003354054	Not displayed	Cellular Growth and Proliferation, Cell Death and Survival, Infectious Disease
LOC100520571	21.369652	0.0006056553	down	XM_003128006	Not displayed	Undetermined
ACTG2	19.070107	0.00027356512	down	NM_001615.3	Not displayed	Inflammatory Disease, Inflammatory Response, Organismal Injury and Abnormalities
TC2N	13.784387	0.008354094	down	NM_028924.3	Supp. Fig. 9	Amino Acid Metabolism, Post-Translational Modification, Small Molecule Biochemistry
*mICSI BCB*^−^ *vs ICSI BCB*^−^ *blastocysts*						
SERPINB10	50.39668	0.0082500875	up	NM_005024.1	Supp. Fig. 12	Cellular Development, Cellular Growth and Proliferation, Hematological System Development and Function
NULL	28.870111	0.008353462	up	unknown	Not displayed	Undetermined
ADAM5	19.022522	0.0037702504	up	XM_003133371	Not displayed	Undetermined
NULL	16.16526	0.002920586	up	unknown	Not displayed	Undetermined
LOC100625749	14.306636	0.005585119	up	XM_003361441	Not displayed	Undetermined
LOC100513964	98.986435	0.000018220118	down	XM_003134882	Not displayed	Undetermined
NULL	50.691635	0.000014981616	down	unknown	Not displayed	Undetermined
IL18	37.155396	0.00052243227	down	NM_001243211	Supp. Fig. 12	Cell-To-Cell Signaling and Interaction, Cell Signaling, Molecular Transport
LOC100623696	36.893543	0.008665602	down	XM_003356823.1	Not displayed	Undetermined
MATN3	35.507694	0.00008970674	down	NM_002381	Supp. Fig. 13	Cell Morphology, Cellular Assembly and Organization, Cellular Development
*mICSI BCB*^−^ *vs ICSI BCB*^*+*^*blastocysts*						
PPP1R9A	57.586193	0.00099	up	NM_001166160	Not displayed	Cellular Compromise, Infectious Disease, Drug Metabolism
NULL	56.806114	0.003806175	up	unknown	Not displayed	Undetermined
LOC100623901	44.763016	0.000384	up	XM_003361640	Not displayed	Undetermined
LOC100526251	38.66665	0.0000941	up	XM_003132878.1	Not displayed	Undetermined
LIX1L	35.739998	0.000215	up	NM_153713	Not displayed	Undetermined
LOC100524253	56.918842	0.007135105	down	XM_003355205	Not displayed	Undetermined
LOC100628218	41.88254	0.002997461	down	XM_003354336.1	Not displayed	Undetermined
ZC3HAV1	40.911594	0.001792942	down	NM_024625	Not displayed	Cellular Compromise, Infectious Disease, Drug Metabolism
NULL	31.49758	0.005764906	down	unknown	Not displayed	Undetermined
NR3C1	21.502375	0.007265344	down	NM_001008481.1	Not displayed	Cellular Compromise, Infectious Disease, Drug Metabolism

**Table 4:** Functional clustering of differentially expressed genes between ICSI BCB^+^ and ICSI BCB^−^; mICSI BCB^−^ and ICSI BCB^−^; and mICSI BCB^−^ and ICSI BCB^+^ blastocysts following unpaired t-tests, with FC > 2 (abs) and significance of p < 0.01.

Clustering of differentially expressed genes (T-Test, p value < 0.01, abs. FC > 2)	Top Diseases and Functions	Network(s)	Predicted upstream Regulator	Molecule Type	Regulation	Activation z-score (abs. Fold-change > 1.5)	p-value of overlap (p < 0.05)
*ICSI BCB*^−^ *vs ICSI BCB*^*+*^*blastocysts*							
BAG3, CCBL1, CGREF1, CREM, EDN1, FAM73A, JUN, KLF5, OXNAD1, PITPNB, SMN1/SMN2	Cellular Assembly and Organization, Cellular Function and Maintenance, Cell Death and Survival	Supp. Figs 7, 8, 9, 12, 17 and 18	CREB1	transcription regulator	Activation	2.617	1.57E-03
BCAT2, EDN1, EPHX2, JUN, MXD3, PGK1, PRDX2, SMTN, SPINT1, UCK1	Cancer, Cellular Movement, Organismal Development	Supp. Figs 7, 8 and 18	ERBB2	kinase	Activation	2.236	1.51E-02
EDN1, JUN, KLF5, SRF	Inflammatory Disease, Inflammatory Response, Organismal Injury and Abnormalities	Supp. Figs 7, 12 and 18	lysophosphatidic acid	chemical - other	Activation	1.958	2.04E-03
CALU, CELF2, CERS4, EPHX2, JUN, LARGE, LTBP1, VAV3	Cancer, Cellular Movement, Organismal Development	Supp. Fig. 7	FOS	transcription regulator	Activation	1.698	3.59E-02
AEBP1, EDN1, JUN, PGK1, PTPN11, SLC25A11	Cell Morphology, Cellular Assembly and Organization, Cellular Function and Maintenance	Supp. Figs 8 and 18	Insulin	hormone	Activation	1.513	3.02E-02
EDN1, JUN, MIOX, PRDX2	Cell To Cell Signaling and Interaction, Reproductive System Development and Function, Cell Signaling	Supp. Fig. 16	N-acetyl-L-cysteine	chemical drug	Inhibition	−1.941	5.0E-02
*mICSI BCB*^−^ *vs ICSI BCB*^−^ *blastocysts*							
CCND2, EDN1, IL18, TRAF2	Cellular Development, Cellular Growth and Proliferation, Hematological System Development and Function	Supp. Figs 8, 12, 17 and 18	Resveratrol	Chemical drug	Activation	1.954	1.19E-02
CCND2, CGREF1, EDN1, IL18, TRAF2	Cellular Development, Cellular Growth and Proliferation, Hematological System Development and Function	Supp. Figs 7, 8, 12, 17 and 18	NFkB (complex)	Complex	Inhibition	−2.208	3.62E-02
CCND2, LIMK2, NABP1, UPB1	Cellular Movement, Hematological System Development and Function, Immune Cell Trafficking	Supp. Figs 11 and 16	IL5	Cytokine	Inhibition	−2.000	9.57E-03
*mICSI BCB*^−^ *vs ICSI BCB*^*+*^*blastocysts*							
ATF4, CLDN7, CYTH2, DDIT4, FTH1, LDHB, MCL1, SMN1/SMN2, TP53I3, UBE2S	Lipid Metabolism, Small Molecule Biochemistry, Vitamin and Mineral Metabolism	Not displayed	MYC	Transcription regulator	Activation	2.156	3.13E-02
AP2A2, ATF4, DPM3, LRRFIP1	Cellular Compromise, Infectious Disease, Drug Metabolism	Supp. Fig. 7	STAT4	Transcription regulator	Activation	2	4.01E-02
ADIPOR2, FABP3, MIOX, STUB1	Carbohydrate Metabolism, Cell Cycle, Developmental Disorder	Supp. Fig. 16	Streptozocin	Chemical drug	Activation	1.98	3.46E-02

DEGs were clustered according to their predicted upstream regulators based on prior knowledge of expected regulation from the Ingenuity^®^ Knowledge Base.
