# Supplementary material for: Restoration of normal embryogenesis by mitochondrial supplementation in pig oocytes exhibiting mitochondrial DNA deficiency
Source: Sci Rep. 2016 Mar 18;6:23229. doi: 10.1038/srep23229 (PMC4796791; doi:10.1038/srep23229)
Supplement: Supplementary Information [file srep23229-s1.pdf]

Supplementary Information

Restoration of normal embryogenesis by mitochondrial supplementation in pig oocytes exhibiting mitochondrial DNA deficiency

Gael LM Cagnone[1,2], Te-Sha Tsai[1,2], Yogeshwar Makanji[1,2], Pamela Matthews[1,2], Jodee Gould[2,3], Michael S. Bonkowski[4], Kirstin D. Elgass[5], Ashley S. A. Wong[6], Lindsay E. Wu[6], Matthew McKenzie[1,2], David A. Sinclair[4] and Justin C. St. John[1,2]

[1] Centre for Genetic Diseases, Hudson Institute of Medical Research, 27-31 Wright Street, Clayton, Vic 3168, AUSTRALIA.

[2] Department of Molecular and Translational Science, Monash University, 27-31 Wright Street, Clayton, Vic 3168, AUSTRALIA.

[3] Centre for Innate Immunity and Infectious Diseases, Hudson Institute of Medical Research, 27-31 Wright Street, Clayton, Vic 3168, AUSTRALIA.

[4] Department of Genetics, Harvard Medical School, 77 Avenue Louis Pasteur, Boston, MA, 02115, USA.

[5] Monash Micro Imaging, Monash University, 27-31 Wright Street, Vic 3168 Clayton, AUSTRALIA.

[6] School of Medical Sciences, University of New South Wales, NSW 2052, AUSTRALIA.

Correspondence should be addressed to JCS (Justin.StJohn@hudson.org.au)

Supplementary Tables:

Supplementary Table 1 Maturation rates for BCB^{+} and BCB^{-} oocytes. **** represents a significant difference of $p<0.0001$.

IVM selection	% MII /unlysed	% lysed oocytes
BCB^{+}	51.4	-
BCB^{-}	20.3****	51.2****

Supplementary Table 2 Differentially expressed genes (T-Test, FDR p0.05).

Differentially expressed genes (T-Test, FDR p0.05)	**BCB- ICSI vs BCB+ ICSI**			
	FC (abs)	Regulation	p (Corr)	Annotation
PARD3	12.798875	up	0.007226747	par-3 family cell polarity regulator
LOC100518872	12.26795	up	0.0063775624	PREDICTED: Sus scrofa cyclin-Y-like protein 1-like
ITIH3	2.560489	down	0.007226747	inter-alpha-trypsin inhibitor heavy chain 3
CACNG2	1.7065419	down	0.009950955	calcium channel, voltage-dependent, gamma subunit 2
LOC100511338	1.6169696	down	0.009950955	putative olfactory receptor 51H1-like [*Sus scrofa* (pig)]

Differentially expressed genes (T-Test, FDR p0.05)	**BCB- mICSI vs BCB- ICSI**			
	FC (abs)	Regulation	p (Corr)	Annotation
RNF185	4.944855	down	0.042696994	ring finger protein 185

Differentially expressed genes (T-Test, FDR p0.05)	**BCB- mICSI vs BCB+ ICSI**			
	FC (abs)	Regulation	p (Corr)	Annotation
TRUB2	11.971094	up	0.022296814	TruB pseudouridine (psi) synthase family member 2
U2	9.439158	down	0.022296814	U2 spliceosomal RNA
Novel Gene	7.371409	up	0.03748932	Uncharacterized
LOC100155709	5.664736	up	0.022296814	PREDICTED: Sus scrofa similar to Ras and Rab interactor 3
LOC100621956	3.0817018	down	0.022296814	Uncharacterized

LOC100514474	1.7616882	down	0.025045672	RNA binding motif protein 25-like [*Sus scrofa*(pig)]
Novel Gene	1.3905554	down	0.025045672	Uncharacterized

Supplementary Table 3 Differentially expressed genes, ANOVA FDR 0.05. Fold-change is relative to BCB$^+$ ICSI.

GENE_SYMBOL	ANOVA FDR	BCB$^-$ ICSI	BCB$^-$ mICSI	REFSEQ	ANNOTATION
		Fold-change / BCB$^+$ ICSI			
LOC100513964	0.001397547	109.53825	1.1065983	XM_003134882	YKT6 v-SNARE homolog (S. cerevisiae) [*Sus scrofa* (pig)]
LOC100155709	0.043959606	4.357983	5.664736	XM_001927068	PREDICTED: Sus scrofa similar to Ras and Rab interactor 3
LOC100511338	0.022049557	-1.6169696	-1.2750927	XM_003129557	Putative olfactory receptor 51H1-like [*Sus scrofa* (pig)]
LOC100621956	0.029266333	-4.254771	-3.0817018	XM_003353158	Uncharacterized
NULL	0.043959606	-1.7189069	-1.3905554	Unknown	Unknown
NULL	0.029266333	-2.3549466	1.3950651	Unknown	Unknown

Supplementary Table 4 Top networks for differentially expressed genes between ICSI BCB$^-$ and ICSI BCB$^+$ blastocysts (378 DEGs, 276 annotated).

Networks	Molecules in Network	Score	Focus Molecules	Top Diseases and Functions
1	Adaptor protein 2, ADRB, Akt, Ap2 alpha, AP2A2, AP2S1, ATG9A, BOK, CGREF1, Ck2, Clathrin, CREM, Cyclin A, Cyclin E, ENDOD1, EP300, EPS15L1, estrogen receptor, KLF5, LTBP1, MXD3, N-cor, NCOR1, NDFIP1, NUMB, OXNAD1, Pias, PPARë±-RXRë±, SAFB2, SENP3, SERCA, SNAP91, SRI, thyroid hormone receptor, TRAP1	39	21	Cellular Assembly and Organization, Cellular Function and Maintenance, Cell Death and Survival
2	Alpha Actinin, Ap1, ARHGEF1, BAG3, FAM118A, IBTK, Ifn, IFN Beta, Ige, IgG, Igm, IL1, Immunoglobulin, Interferon alpha, KIRREL, MAPKAPK3, NFkB (complex), NOXO1, OAS1, PDLIM2, PGK1, PLC gamma, PRKCSH, Pro-inflammatory Cytokine, PTPN11, ROCK2, Sapk, SPINT1, STK16, TCR, TFG, TMEM92, Tnf receptor, TRAF2, WDR34	34	19	Cell Morphology, Cellular Assembly and Organization, Cellular Function and Maintenance
3	AAMDC, APIP, APRT, ATAD1, BAAT, CCDC120, CYTH2, DSC2, ELAVL1, EVPL, FAM178A, FAM73A, FBXO31, GALNT1, HNF4A, LMAN2L, MAPK3, MRPL37, NAGPA, NUDT11, NUFIP1, PEX3, PEX13, RAPH1, RTFDC1, SPATA6, STARD7, TANGO2, TC2N, TMEM189, TRIM15, TRUB2, UBC, YIPF2, ZNF300	32	18	Amino Acid Metabolism, Post-Translational Modification, Small Molecule Biochemistry
4	ABHD4, ARF5, ATL3, BLOC1S2, CDS1, COMMD3, COMMD4, COMMD6, EML3, HEYL, HIST1H2BL, INIP, KATNA1, KATNB1, KBTBD6, LEPRE1, LMTK2, LRRC41, LZTS2, MBOAT7, NABP1, OSBPL3, OSBPL9, OSBPL10, OSBPL11, RAB11FIP4, RAB11FIP5, RACGAP1, RALB, RGL3, RHOBTB1, SMPD4, SMTN, UBC, YIF1A	27	16	Cancer, Cell Death and Survival, Cell Morphology
5	AEBP1, Alpha actin, CERS4, COL17A1, collagen, Collagen(s), DBNL, DDR2, DIO3, ECSIT, EDN1, ERK1/2, Hsp27, INSL6, ITIH3, JINK1/2, LARGE, Mek, NADPH oxidase, NFAT (complex), Pdgf (complex), PDGFA, Pkg, PRDX2, PRDX5, PTPase, Rar, Rock, Rsk, SEMA3F, Smad, Sos, Tgf beta, TSH, VAV3	27	16	Cancer, Cellular Movement, Organismal Development
6	AMDHD2, APOOL, BRD4, C16orf59, CEBPD, CHID1, CRKL, DAZAP2, DHRS13, EHMT1, FAM178A, FAM203A, FAR1, FEZ1, FEZ2, GALK1, GALK2, GRTP1, HIST3H3, KANK4, LIMD2, MGEA5, OPTN, PLBD2, PSMA1, PSMD9, SETD8, SLC44A2, SMYD3, TERT, TRIT1, UBC, WHSC1, ZFAND2B, ZNF709	25	15	Cellular Growth and Proliferation, Cell Death and Survival, Infectious Disease
7	AAMDC, ABCF1, APP, ARAP1, ARNT2, BCAT2, CCBL1, CDH2, CELF2, CTNNA1, DLGAP5, DRAP1, ESR2, EXOSC3, FCHO1, GORASP2, GSTA3, HIST1H2AD, HTRA2, KIF3B, KIF5C, NUMB, PDCD7, PDSS2, PHYHD1, POLE3, PTPRQ, Rab11, ST6GALNAC6, STAU1, SUSD4, TAF9B, TCP11,	24	15	Cellular Assembly and Organization, Cellular Function and Maintenance, Cardiovascular System Development and Function

	THY1, TNNT2			
8	ACTG2, Actin, ANKRA2, ATP6V0D2, calpain, CaMKII, CD3, Cofilin, Collagen type I, COPB2, Creb, ERK, F Actin, Growth hormone, Gsk3, GTF3C1, Lh, LSM7, MAP2K1/2, Nfat (family), NSMF, PDGF BB, PI3K (complex), PITPNB, Pkc(s), Rac, Ras homolog, RNF10, SLC25A11, SMN1/SMN2, SRF, STAT5a/b, UBE2W, UCK1, WDR26	22	15	Inflammatory Disease, Inflammatory Response, Organismal Injury and Abnormalities
9	ANP32E, ATAD1, ATG4A, CACHD1, CALU, CARHSP1, CBX8, CTSF, CYP1A1, DPCD, DYNC2H1, FRMD4A, IKBIP, MLLT1, NUP43, PARD3, PCGF5, PDRG1, PDZD11, PEX3, PEX7, PEX10, PEX13, PEX14, PHAX, PNMAL1, PPP6R2, REEP5, SNORA70, TAF1C, TIPRL, TP53, TRIAP1, TUBB8, UBC	22	14	Developmental Disorder, Hereditary Disorder, Metabolic Disease
10	26s Proteasome, ABCC2, caspase, CD93, CELF2, Cg, CSNK1A1, DSG3, EPHX2, Focal adhesion kinase, FSH, GNRH, Hdac, hemoglobin, Histone h3, Histone h4, Insulin, Jnk, JUN, Mapk, Mmp, P38 MAPK, PEMT, Pka, Ras, RNA polymerase II, SMAD4, Smad2/3, TBX3, THRAP3, TRAF1-TRAF2-TRAF3, Ubiquitin, UNC45A, Vegf, WDR36	18	12	Cardiovascular System Development and Function, Cellular Development, Skeletal and Muscular System Development and Function
11	ABCB11, beta-estradiol, betaine, CALCRL, CNN2, CRK, cyclic AMP, CYSTM1, DOK7, EVX1, F2RL2, GLRA1, GLRA2, GLRA3, GLRA4, GLRB, GRM4, HACE1, HOXD4, KMT2B, KRT1, LPAR4, MIOX, myo-inositol, OPRK1, PRICKLE3, RAMP2, RAMP3, RARB, Relaxin, SLC35D3, SLC6A6, taurine, TRHR, VIPR1	11	8	Cell-To-Cell Signaling and Interaction, Reproductive System Development and Function, Cell Signaling
12	C17orf97, JMJD6	2	1	Organismal Development, Developmental Disorder, Ophthalmic Disease

Supplementary Table 5 Predicted up-stream regulators for differentially expressed genes between ICSI BCB^{-} and ICSI BCB^{+} blastocysts.

**Upstream Regulator**	**Molecule Type**	**Predicted Activation State**	**Activation z-score**	**p-value of overlap**	**Target molecules in dataset**
CREB1	Transcription regulator	Activated	2.617	1.57E-03	↑BAG3, ↑CCBL1, ↑CGREF1, ↓CREM, ↑EDN1, ↓FAM73A, ↑JUN, ↑KLF5, ↑OXNAD1, ↓PITPN
ERBB2	Kinase	Activated	2.236	1.51E-02	↑BCAT2, ↑EDN1, ↑EPHX2, ↑JUN, ↑MXD3, ↑PGK1, ↑PRDX2, ↑SMTN, ↑SPINT1, ↑UCK1
BMP2	Growth factor	Activated	2.000	5.24E-02	↑EPHX2, ↑JUN, ↓SMAD4, ↑SPINT1
troglitazone	Chemical drug	Activated	2.000	2.90E-01	↑JUN, ↑PEMT, ↑PTPN11, ↑UCK1
lysophosphatidic acid	Chemical - other		1.958	2.04E-03	↑EDN1, ↑JUN, ↑KLF5, ↑SRF
trichostatin A	Chemical drug		1.938	3.63E-01	↑CGREF1, ↓CREM, ↓SMAD4, ↑SMN1/SMN2, ↑SRF
IL4	Cytokine		1.890	1.61E-01	↑APRT, ↑BCAT2, ↑CELF2, ↑JUN, ↑MAPKAPK3, ↑NABP1, ↑PEX14, ↓VIPR1
FOS	Transcription regulator		1.698	3.59E-02	↓CALU, ↑CELF2, ↑CERS4, ↑EPHX2, ↑JUN, ↑LARGE, ↓LTBP1, ↑VAV3
Insulin	Group		1.513	3.02E-02	↓AEBP1, ↑EDN1, ↑JUN, ↑PGK1, ↑PTPN11, ↑SLC25A11

Supplementary Table 6 Top networks for differentially expressed genes between mICSI BCB$^-$ and ICSI BCB$^-$ blastocysts (192 DEGs, 127 annotated).

Networks	Molecules in Network	Score	Focus Molecules	Top Diseases and Functions
1	ADAMTS5, APP, ASCC1, BACE2, CCND1, COPS5, CTSE, CXCL16, DEFA1 (includes others), ENDOD1, ERBB2, EVPL, EXOC7, FBXO31, IL5, IRF6, KLHL42, LFNG, MANF, MID2, NABP1, OLFML2A, PCIF1, PLBD1, PLS1, PPP6R2, RNF138, RNF185, SGOL1, SHMT2, SMARCD2, SRPX, UBC, UPB1, YKT6	37	18	Cellular Movement, Hematological System Development and Function, Immune Cell Trafficking
2	AFF4, APLP2, BCR (complex), caspase, CCND2, CD3, CDK16, CGREF1, Cyclin A, DNAJA3, ERK1/2, Growth hormone, Hsp27, Ifn gamma, Igm, IL18, Immunoglobulin, KLF5, LEMD3, LNX2, MAP2K1/2, Mek, NECAP2, Raf, Rap1, RAPGEF1, SERPINB10, Sos, STAT5a/b, STXBP4, TCR, Tnf (family), TNFSF9, TOM1L1, TRAF2	36	17	Cellular Development, Cellular Growth and Proliferation, Hematological System Development and Function
3	ADAM19, ADAMTS4, ADAMTS5, BRCC3, C16orf59, CAMSAP1, CDC42BPA, DKC1, DMD, DOCK1, DOCK4, DTNB, ECEL1, ELMO2, ELMO3, FAM172A, GALK2, GALNT2, IRF6, MANF, MARK2, MATN3, MATN4, PIGU, PYGM, RCSD1, RHOG, SLC9A6, SNORA62, SPTBN1, SYNM, TSC22D2, TTC32, UBC, ZC3H7A	25	13	Cell Morphology, Cellular Assembly and Organization, Cellular Development
	AIM2, CD83, CUL4A, CXCL16, EPS15, geranylgeranyl pyrophosphate, Histone h3, Histone h4, Hsp70, IBTK, Il12 receptor, Il18r, IL18R1, IL18RAP, IL36B, Interferon alpha, Jnk, MEFV, NFkB (complex), PI3K (complex), Pkc(s), PPARGC1B, PRDX3, Ras homolog, RHOF, RNA polymerase II, RNF11, RTKN, SMYD3, SPATS2L, THRAP3, TLR1, TRAF1-TRAF2-TRAF3, voltage-gated calcium channel, WSB1	20	11	Cellular Assembly and Organization, Cellular Development, Cellular Growth and Proliferation
5	BDP1, CCR4, CCR5, CFTR, chemokine, CLEC7A, CXCL16, CXCL17, CYSLTR2, DUSP14, FFAR2, glycosaminoglycan, Gpcr, GPR34, GPR132, GPR143, IL18R1, IL18RAP, IL1B, IL36B, IRAK, LHX8, LPAR2, MAPK8, MAS1, MIOX, NFKBIZ, NMBR, OSBPL1A, PKD1, PSEN1, TACR1, TET2, XCL1, ZDHHC17	13	8	Cell-To-Cell Signaling and Interaction, Cell Signaling, Molecular Transport
6	Akt, AMPK, Ap1, ARHGEF12, CaMKII, Collagen type I, Collagen(s), EDN1, ERK, estrogen receptor, Filamin, Focal adhesion kinase, FSH, GRAMD1B, IgG, IL1, Insulin, Lh, LIMK2, Mapk, MAPK1, MEP1B, Mmp, P38 MAPK, Pdgf (complex), PDGF BB, Pro-inflammatory Cytokine, Ras, Rock, Shc, SRC (family), STXBP3, Tgf beta, VAPB, Vegf	13	8	Cellular Assembly and Organization, Cancer, Tumor Morphology
7	DNMT3B, LACC1	2	1	DNA Replication, Recombination, and Repair, Gene Expression, Cancer

Supplementary Table 7 Predicted up-stream regulators for differentially expressed genes between mICSI BCB^{-} and ICSI BCB^{-} blastocysts.

Upstream Regulator	Molecule Type	Predicted Activation State	Activation z-score	Notes	p-value of overlap	Target molecules in dataset
NFkB (complex)	Complex	Inhibited	-2.208	bias	3.62E-02	↓CCND2, ↓CGREF1, ↓EDN1, ↓IL18, ↓TRAF2
IL5	Cytokine	Inhibited	-2.000	bias	9.57E-03	↓CCND2, ↓LIMK2, ↓NABP1, ↓UPB1
HRAS	Enzyme		-1.982		9.89E-02	↓CCND2, ↓CGREF1, ↓KLF5, ↓MAPK1
IL2	Cytokine		-1.406	bias	2.03E-02	↓CCND2, ↓EDN1, ↓IL18, ↑TNFSF9, ↓TRAF2
AGT	Growth factor		-1.114	bias	1.12E-02	↓CCND2, ↓CGREF1, ↓EDN1, ↓IL18, ↓KLF5
EGF	Growth factor		-1.098	bias	3.72E-03	↓CCND2, ↓EDN1, ↓EPS15, ↓LIMK2, ↓MAPK1, ↓TRAF2
IGF1	Growth factor		-1.091	bias	3.64E-02	↓CCND2, ↓EDN1, ↓IL18, ↓MAPK1
Salmonella enterica serotype	Chemical toxicant		-1.000	bias	2.11E-03	↓EDN1, ↓IL18, ↑TNFSF9, ↓UPB1
IL15	Cytokine		-0.900	bias	2.62E-02	↓CCND2, ↓EDN1, ↓MAPK1, ↑PPARGC1B
NKX2-3	Transcription regulator		-0.854		4.43E-03	↓EDN1, ↓FAM172A, ↓SHMT2, ↓SRPX
FOXO1	Transcription regulator		-0.447	bias	3.29E-03	↓IL18, ↓LEMD3, ↑PPARGC1B, ↑TNFSF9, ↓TRAF2
MAP3K8	Kinase		0.000		8.12E-05	↓IL18, ↑PPARGC1B, ↑RCSD1, ↓RHOF, ↓SPATS2L
forskolin	Chemical toxicant		0.000		3.71E-02	↓CCND2, ↓CDK16, ↓EDN1, ↓MAPK1, ↓TRAF2
CD3	Complex		0.928	bias	1.53E-02	↓CCND2, ↑CUL4A, ↓MAPK1, ↑SERPINB10, ↓TOM1L1, ↓TRAF2
U0126	Chemical – kinase inhibitor		1.213	bias	1.58E-02	↓CCNDS, ↓EDN1, ↓GRAMD1B, ↓KLF5, ↓MAPK1
resveratrol	Chemical drug		1.954		1.19E-02	↓CCND2, ↓EDN1, ↓IL18, ↓TRAF2
MXD1	Transcription				4.40E-04	↓CCND2, ↓EDN1

	regulator					
levamisole	Chemical drug				4.40E-04	↓EDN1, ↓IL18
ROCK1	Kinase				7.58E-04	↓CCND2, ↓EDN1
PD 169316	Chemical – kinase inhibitor				1.31E-03	↓CCND2, ↑PPARGC1B
DOCK8	Other				1.38E-03	↓CCND2, ↓EDN1, ↓IL18
SASH1	Other				1.50E-03	↓CCND2, ↓EDN1, ↓IL18
MET	Kinase				1.83E-03	↓CCND2, ↓EDN1, ↓IL18
stearic acid	Chemical – endogenous mammalian				2.01E-03	↓EDN1, ↑PPARGC1B
EPO	Cytokine				2.04E-03	↓CCND2, ↓EDN1, ↓IL18, ↓OSBPL1A, ↑SMARCD2
COL18A1	Other				2.87E-03	↓EDN1, ↓IL18, ↓MAPK1
SAMSN1	Other				3.05E-03	↓CCND2, ↓EDN1, ↓IL18
1D-chiro-inositol	Chemical – endogenous mammalian				3.17E-03	↓MIOX
Sn-glycero-3-phosphocholin	Chemical – endogenous mammalian				3.17E-03	↓MIOX
KRT1	Other				3.17E-03	↓IL18
PHIP	Other				3.17E-03	↓CCND2
PSMC4	Peptidase				3.17E-03	↓CCND2
PSMA2	Peptidase				3.17E-03	↓CCND2
ZKSCAN3	Transcription regulator				3.17E-03	↓CCND2
PSMD6	Enzyme				3.17E-03	↓CCND2
CXXC4	Other				3.17E-03	↓TET2
2.5-dihydroxymethylcinnam	Chemical – kinase inhibitor				3.17E-03	↓EDN1

Supplementary Table 8 Top networks for differentially expressed genes between mICSI BCB$^-$ and ICSI BCB$^+$ blastocysts (311 DEGs, 222 annotated).

Networks	Molecules in Network	Score	Focus Molecules	Top Diseases and Functions
1	Akt, BRD8, CARM1, Cbp/p300, CCNK, CLK1, estrogen receptor, ETV6, FLAD1, GTF2H1, Hdac, HDAC11, HISTONE, Histone h3, Histone h4, Holo RNA polymerase II, IRF3, KAT2B, MEF2, METTL8, N-cor, NCOR1, OSTF1, OXNAD1, PBX3, PHF19, RNA polymerase II, SAFB2, SMARCD2, SMN1/SMN2, SMYD3, SUPT16H, SWI-SNF, TESK1, USP18	43	23	Cell Cycle, DNA Replication, Recombination, and Repair, Cancer
2	26s Proteasome, AFF4, ARHGEF1, ATF4, BCKDHA, Ck2, CSNK1A1, F Actin, FSH, FTH1, FTMT, GMFB, GNGT1, GNRH, Hsp70, Hsp90, IFN Beta, Immunoglobulin, Interferon alpha, LRRFIP1, NFkB (complex), NR3C1, P38 MAPK, PHF3, PI3K (complex), Pka, PKN3, PPP1R9A, STUB1, TP53I3, TRA2A, TUFM, UNC45A, ZC3HAV1, ZFP91	41	21	Cellular Compromise, Infectious Disease, Drug Metabolism
3	ABHD5, ADCK3, ANAPC15, APRT, AS3MT, ASNA1, C8orf4, CHIC2, EEF1D, ELAVL1, EPB41L4B, FAM73A, FASTKD2, GOLGA1, GOPC, HNF4A, IFT122, LIN54, LRRC8A, MRPL37, MUTYH, PDCL3, POGK, PXDC1, RAB6A, S100A14, SLC19A2, SLC27A4, SLC38A1, SLC43A1, TANGO2, TRUB2, UBC, UBLCP1, WDFY3	31	17	Dermatological Diseases and Conditions, Developmental Disorder, Hereditary Disorder
4	AP2A2, APP, ARAP1, ARF4, C11orf63, CDKN1A, CPNE6, DCLK3, DNAH1, DNAH5, DNAH6, DNAH8, DNAH10, DNAH12, DNAH14, DNAH17, DNAI1, DNAI2, DNAL1, DNALI1, DYNLT1, GPKOW, LDHB, MPG, NECAP1, PCIF1, PDS5B, PHYHD1, PP2A, PPM1J, PPP1R2, SCYL1, TFPT, TIPRL, UBE2S	29	17	Developmental Disorder, Hereditary Disorder, Respiratory Disease
5	ARMC8, ARV1, ATF7IP, COMMD3, COMMD4, COMMD6, CPPED1, DERL1, DOCK1, ELMO1, ELMO2, ELMO3, FBXO44, GID8, KIAA0368, LEPREL4, LIN28B, MAEA, MAGEA11, MCAT, MKLN1, MTERF4, NPLOC4, NSUN4, PCDHB4, PTDSS1, RANBP9, RMND5A, SLC30A5, SLC30A6, SUSD1, TRUB2, UBC, ZFAND2B, ZNF777	28	16	Cancer, Cellular Movement, Neurological Disease
6	APPBP2, ARHGEF3, AUH, AURKAIP1, BDP1, CELF2, CEP131, DCAF6, DCAF8, DMPK, DUSP22, ENC1, ETFB, FGFR1OP2, GEMIN5, INPP5K, LGALS8, MAPK8, MBOAT7, MIOX, MZB1, NFE2L2, OAZ1, OSGIN1, PANK2, PHAX, POMT1, RAB11FIP4, RAB11FIP5, RAC3, SCG2, SCOC, SPRED2, UBC, VPS36	24	14	Endocrine System Development and Function, PostOTranslational Modification, Developmental Disorder
7	14-3-3, Ap1, ATP1A3, Collagen(s), Creb, CYTH2, DBNL, DCN, DDIT4, Dynamin, ELK1, ERK1/2, GNAI2, HSH2D, IARS, Igm, IL1, ITSN1, LDL, MAP2K1/2, MCL1, Mek, Nfat (family), p70 S6k, p85 (pik3r), Pdgf (complex), PDGF BB, PELI1, Pias, Ras, RBM7, TCR, TEAD4, Tgf beta, XPNPEP2	23	15	Cellular Assembly and Organization, Nervous System Development and Function, Antigen Presentation
8	ADA, ARHGEF2, CLDN7, CSF2, CYP11B1, CYP1A1, dolichyl-phosphate	15	10	Lipid Metabolism, Small Molecule

	beta-D-mannosyltransferase, DPM2, DPM3, EGF, Eif4g, GAS1, GIGYF2, GSTA1, Importin alpha, LTB, Nuclear factor 1, PDE4B, PDE6A, PP1 protein complex group, Ppp1cc, PPP1R12C, PQLC3, PURA, RMDN3, SLC37A1, SNORA70, SNTB1, SOX4, SRC (family), TJP2, TNFRSF9, TP53, TRA2B, YWHAZ			Biochemistry, Vitamin and Mineral Metabolism
9	Actin, ADIPOR2, CD3, CLCF1, CPEB2, DDAH2, DUSP16, DUSP22, Eif4g, ERK, FABP3, Fgfr, IL1RAP, IL36G, IL3RA, Insulin, Jnk, JUN/JUNB/JUND, MAP4K3, Mapk, mir-210, miR-146a-5p (and other miRNAs w/seed GAGAACU), NAGA, PITPNB, Pkc(s), PLEKHA4, PP1 protein complex group, PRC1, PSMC3IP, PTP4A2, SPRED2, TLR1, TMSB10/TMSB4X, VAV, Vegf	10	7	Carbohydrate Metabolism, Cell Cycle, Developmental Disorder
10	ADAL, adenosine deaminase	2	1	Infectious Disease, Reproductive System Disease, Cancer

Supplementary Table 9 Predicted up-stream regulators for differentially expressed genes between mICSI BCB$^-$ and ICSI BCB$^+$ blastocysts.

Upstream Regulator	Molecule Type	Predicted Activation State	Activation z-score	Notes	p-value of overlap	Target molecules in dataset
methylprednisolone	Chemical drug	Activated	2.219		9.84E-02	↑AP2A2, ↑CLDN7, ↑DDIT4, ↓GNAI2, ↓NR3C1, ↑SMN1/SMN2
MYC	Transcription regulator	Activated	2.156		3.13E-02	↑ATF4, ↑CLDN7, ↑CYTH2, ↑DDIT4, ↑FTH1, ↑LDHB, ↓MCL1, ↑SMN1//SMN2, ↑TP5313, ↑UBE2S
STAT4	Transcription regulator	Activated	2.000	bias	4.01E-02	↑AP2A2, ↑ATF4, ↑DPM3, ↑LRRFIP1
streptozocin	Chemical drug		1.980		3.46E-02	↑ADIPOR2, ↑FABP3, ↑MIOX, ↑STUB1
TP73	Transcription regulator		1.940		9.37E-02	↑AUH, ↑DDIT4, ↑MUTYH, ↑TP5313
troglitazone	Chemical drug		1.432		1.90E-03	↓ARF4, ↑BCKDHA, ↓DCN, ↑ELK1, ↑FABP3, ↓MCL1, ↑PCIF1, ↑USP18
etoposide	Chemical drug		1.412		1.24E-02	↑DDIT4, ↑FTH1, ↓MCL1, ↑TP5313
FGF2	Growth factor		1.408		1.59E-02	↑ATF4, ↓DCN, ↑ELK1, ↑FTH1, ↓NR3C1, ↑TEAD4
miR-16-5p (and other miRNAs w/seed AGCAGCA)	Mature microrna		1.214	bias	2.77E-02	↓GTF2H1, ↓MCL1, ↓PURA, ↑UBE2S
CREB1	Transcription regulator		1.000		3.74E-02	↓ETV4, ↓FAM73A, ↓MCL1, ↑OSTF1, ↑OXNAD1, ↓PITPNB, ↑SMN1/SMN2
dexamethasone	Chemical drug		0.768		4.07E-04	↑ATF4, ↑AUH, ↑BCKDHA, ↑CELF2, ↓CSNK1A1, ↓DCN, ↑DDIT4, ↑ELK1, ↑FTH1, ↓GNAI2
N-acetyl-L-cysteine	Chemical drug		0.277		2.77E-02	↑ATF4, ↑ATP1A3, ↓GNAI2, ↑MIOX
BRCA1	Transcription regulator		0.152		1.05E-02	↑DDIT4, ↓GTF2H1, ↓NR3C1, ↑TP53I3
thapsigargin	Chemical toxicant		0.137		1.50E-02	↑ADIPOR2, ↑ATF4, ↑DDIT4,

						↓MCL1
tunicamycin	Chemical – endogenous non-mammalian		-0.045		9.32E-03	↑ADIPOR2, ↑ATF4, ↑DDIT4, ↓MCL1
D-glucose	Chemical – endogenous mammalian		-0.065		2.94E-02	↓ARF4, ↑ATF4, ↓DCN, ↑DDIT4, ↑MIOX, ↓NR3C1, ↑STUB1
valproic acid	Chemical drug		-0.176		6.90E-03	↓ABHD5, ↑CARM1, ↑DPM2, ↓ETV6, ↓GNAI2, ↓NR3C1, ↑SMN1/SMN2
phorbol myristate acetate	chemical drug		-0.258		1.72E-03	↑APRT, ↓ARF4, ↑ATP1A3, ↑DDIT4, ↑EEF1D, ↑ELK1, ↑FTH1, ↓GNAI2, ↓HSH2D, ↓KAT2B
IRF7	Transcription regulator		-0.277	bias	2.92E-03	↑ATF4, ↓MCL1, ↓PELI1, ↑USP18, ↓ZC3HAV1
Benzyloxycarbonyl-Leu-Leu-Leu aldehyde	Chemical – protease inhibitor		-0.278		2.21E-02	↑ATF4, ↓DCN, ↓MCL1, ↑NCOR1, ↓NR3C1
sirolimus	Chemical drug		-0.647		3.62E-03	↑ATF4, ↑BCKDHA, ↑DDIT4, ↑FTH1, ↑LDHB, ↓MCL1, ↓NR3C1, ↑TUFM
5-fluorouracil	Chemical drug		-1.067		4.36E-02	↑ATF4, ↑IARS, ↓MCL1, ↑PRC1
camptothecin	Chemical reagent		-1.387		9.05E-04	↑ARHGEF1, ↑CYTH2, ↓GNAI2, ↑ITSN1, ↑MBOAT7, ↓MCL1, ↓NR3C1, ↑OSTF1, ↑TP53I3 ↓TRA2A

Supplementary Table 10 Top networks for genes that are similar between ICSI BCB$^+$ and mICSI BCB$^-$ but different to ICSI BCB$^-$ blastocysts (168 genes identified, 90 annotated)

Networks	Molecules in Network	Score	Focus Molecules	Top Diseases and Functions
1	BACE2, C11orf49, C9orf40, DGKE, DLEC1, ENDOD1, EPS15L1, EVPL, FAS, FN1, GABRA5, GGA1, GIPC3, HPS4, hydrogen peroxide, KLHL42, LRP3, MBOAT7, METTL2A, MIOX, NABP1, NNT, NUP43, PLAG1, PPP6R2, PTP4A3, RAC1, SHMT2, SLC7A8, STAB1, STAT3, UBC, UBR2, ZNF419, ZNF860	48	22	Cell Morphology, Connective Tissue Development and Function, Cell Death and Survival
2	26s Proteasome, ACADS, Actin, Akt, Ap1, ASCC1, caspase, CCDC120, CD3, CHAF1A, ERK, Histone h3, Histone h4, Hsp90, IBTK, IKK (complex), Insulin, LRWD1, MID2, MYBPC1, NCK1, NFkB (complex), NR1I2, PARK2, PCCB, PITPNM1, PLS1, Proinsulin, Rac, SMARCE1, THRAP3, TRAF2, UBE2E2, UBN1, Vegf	40	19	Gene Expression, Protein Synthesis, Connective Tissue Disorders
3	APLP2, ATP13A2, CDK16, CGREF1, Creb, Cyclin E, EDN1, ERBB2, ERK1/2, estrogen receptor, FKBP5, FSH, Growth hormone, GTPase, KLF5, Ldh (complex), Lh, Mapk, Mek, N-cor, NOD1, PER1, Pkc(s), POU1F1, PRCC, RABAC1, Ras, RASAL2, RGS12, ROCK2, Rsk, Sos, TNIP1, Ubiquitin, WDYHV1	37	18	Cell-To-Cell Signaling and Interaction, Cellular Assembly and Organization, Cell Cycle
4	ADARB1, ALB, C16orf59, C4orf51, CA9, CLOCK, CSNK2A2, DGAT2, DYNLL1, FAM172A, FAM178A, FEZ1, FEZ2, FOXR1, GRK5, GSTK1, GSTP1, HEATR3, MGAT2, OPTN, PIGU, PLBD2, PPCDC, PSMA1, SCAF1, SDAD1, SLC25A32, TGIF1, TMEM63B, TOR4A, TVP23B, TXN2, UBC, UBQLN4, WHSC1	30	15	Lipid Metabolism, Small Molecule Biochemistry, Cellular Development
5	ARHGEF12, CD4, EFNB3, FBXO31, FFAR2, Gzmb, HNF4α dimer, IL1B, JAM2, Jnk, MAP3K6, MAP4K2, Mapk kinase, MAS1, MTSS1, OCLN, P38 MAPK, PARD3, Pka, PLN, progesterone, RHOC, RHOQ, SLN, SMPDL3A, TAB3, TET2, TMEM79, TNFRSF17, Tnfrsf22/Tnfrsf23, TNFSF9, TNFSF15, TNMD, TRAF1-TRAF2-TRAF3, UBL7	11	7	Cellular Development, Cellular Growth and Proliferation, Hematological System Development and Function

Supplementary Table 11 Predicted upstream regulators of genes that are commonly expressed between ICSI BCB^{+} and mICSI BCB^{-} but different to ICSI BCB^{-} blastocysts.

Upstream Regulator	Molecule Type	Predicted Activation State	Activation z-score	Notes	p-value of overlap	Target molecules in dataset
HR	transcription regulator				4.97E-04	↓DLEC1, ↑IBTK, ↑UBR2
forskolin	chemical toxicant		-0.568	bias	6.54E-04	↓CDK16, ↓CGREF1, ↓EDN1, ↑FKBP5, ↓PER1, ↓POU1F1, ↓RASAL2, ↓SLC7A8, ↓TRAF2
fish oils	chemical drug				7.37E-04	↓EDN1, ↓KLF5, ↓NR1I2
TFAP2B	transcription regulator				1.18E-03	↓EDN1, ↓ERBB2
Lh	complex				2.19E-03	↓CDK16, ↑FKBP5, ↓PER1, ↓RASAL2, ↓RGS12
methylselenic acid	chemical reagent				2.31E-03	↓CDK16, ↓CHAF1A, ↑FKBP5, ↓RABAC1, ↓SMARCE1
lysophosphatidic acid	chemical - other				2.68E-03	↓EDN1, ↑FKBP5, ↓KLF5
1D-chiro-inositol	chemical - endogenous mammalian				3.68E-03	↓MIOX
sn-glycero-3-phosphocholine	chemical - endogenous mammalian				3.68E-03	↓MIOX
tandospirone	chemical drug				3.68E-03	↓PER1
CLEC16A	other				3.68E-03	↑PARK2
miR-548d-3p (and other miRNAs w/seed AAAAACC)	mature microrna				3.68E-03	↓ERBB2
TIMELESS	other				3.68E-03	↓PER1
LINC00570	other				3.68E-03	↓ROCK2
SMARCC2	transcription regulator				3.68E-03	↓SMARCE1
DECR1	enzyme				3.68E-03	↓ERBB2
CXXC4	other				3.68E-03	↓TET2
SR11256	chemical reagent				3.68E-03	↓EDN1

2,5-dihydroxymethylcinnamate	chemical - kinase inhibitor				3.68E-03	↓EDN1
triazolam	chemical drug				3.68E-03	↓PER1
tartaric acid	chemical reagent				3.68E-03	↓ERBB2
clorobiocin	chemical drug				7.34E-03	↓ERBB2
myo-inositol	chemical - endogenous mammalian				7.34E-03	↓MIOX
retaspimycin	chemical drug				7.34E-03	↓ERBB2
silipide	chemical drug				7.34E-03	↓ERBB2
RBM17	other				7.34E-03	↓ERBB2
LRIG3	other				7.34E-03	↓ERBB2
Trk Receptor	group				7.34E-03	↓EDN1
ACPP	phosphatase				7.34E-03	↓ERBB2
miR-548h-5p (and other miRNAs w/seed AAAGUAA)	mature microrna				7.34E-03	↓ERBB2
ECE1	peptidase				7.34E-03	↓EDN1
SLC8A1	transporter				7.34E-03	↓EDN1
WWP1	enzyme				7.34E-03	↓KLF5
EEF2K	kinase				7.34E-03	↓KLF5
PLCD4	enzyme				7.34E-03	↓ERBB2
Bhlhe41	transcription regulator				7.34E-03	↓PER1
BIIB028	chemical drug				7.34E-03	↓ERBB2
lavendustin A	chemical - kinase inhibitor				7.34E-03	↓EDN1
phosphoramidon	chemical - endogenous non-mammalian				7.34E-03	↓EDN1
coumermycin	chemical drug				7.34E-03	↓ERBB2
GnRH analog	biologic drug		0.447		7.92E-03	↓CDK16, ↓NR1I2, ↓PRCC, ↓SMARCE1, ↓TRAF2
aldosterone	chemical - endogenous mammalian				8.61E-03	↓EDN1, ↑FKBP5, ↓POU1F1
histone deacetylase	complex				9.07E-03	↓PER1, ↓SLC7A8

emodin	chemical drug				9.99E-03	↓ERBB2, ↓TRAF2
LIPE	enzyme				1.06E-02	↓EDN1, ↑FKBP5, ↓PER1
ATF4	transcription regulator				1.06E-02	↓EDN1, ↑PARK2, ↓SHMT2
Dexamethasone-GR	complex				1.10E-02	↓NR1I2
muraglitazar	chemical drug				1.10E-02	↓EDN1
DDX54	transcription regulator				1.10E-02	↓ERBB2
CSNK1E	kinase				1.10E-02	↓PER1
miR-331-3p (miRNAs w/seed CCCCUGG)	mature microrna				1.10E-02	↓ERBB2
URI1	transcription regulator				1.10E-02	↑FKBP5
AKAP13	other				1.10E-02	↑FKBP5
VEZF1	transcription regulator				1.10E-02	↓EDN1
Mn2+	chemical - endogenous mammalian				1.10E-02	↓ATP13A2
TNF	cytokine		-0.234	bias	1.14E-02	↓ACADS, ↓EDN1, ↓ERBB2, ↓GABRA5, ↓KLF5, ↓NCK1, ↓NR1I2, ↓SLC7A8, ↓SMPDL3A, ↓TNIP1, ↓TRAF2, ↑UBR2
PGR	ligand-dependent nuclear receptor				1.21E-02	↓ERBB2, ↑FKBP5, ↓KLF5, ↓MBOAT7
epicatechin	chemical drug				1.25E-02	↑NUP43, ↓PITPNM1
luminespib	chemical drug				1.46E-02	↓ERBB2
WBSCR22	enzyme				1.46E-02	↑FKBP5
(±)-2-hydroxyoleic acid	chemical drug				1.46E-02	↓ROCK2
AES	transcription regulator				1.46E-02	↓POU1F1
RGD1560225	other				1.46E-02	↓EDN1
LPAR3	g-protein coupled receptor				1.46E-02	↓KLF5
N-hydroxy-2,2-diphenylacetamide	chemical reagent				1.46E-02	↓EDN1
nandrolone decanoate	chemical drug				1.46E-02	↓GABRA5

testosterone cypionate	chemical drug				1.46E-02	↓GABRA5
Go 6976	chemical - kinase inhibitor				1.52E-02	↓ERBB2, ↓TRAF2
KLF5	transcription regulator				1.63E-02	↓CGREF1, ↓KLF5
MED1	transcription regulator				1.73E-02	↓CHAF1A, ↓ERBB2, ↓TET2
CLOCK	transcription regulator				1.73E-02	↑FKBP5, ↑MGAT2, ↓PER1
GHRL	growth factor				1.81E-02	↓EDN1, ↓POU1F1
darusentan	chemical drug				1.83E-02	↓EDN1
Gata	group				1.83E-02	↓EDN1
miR-148a-3p (and other miRNAs w/seed CAGUGCA)	mature microrna				1.83E-02	↓NR1I2
CITED1	transcription regulator				1.83E-02	↓ERBB2
GPR37	g-protein coupled receptor				1.83E-02	↑PARK2
miconazole	chemical drug				1.83E-02	↓NR1I2
methyltestosterone	chemical drug				1.83E-02	↓GABRA5
vorinostat	chemical drug				1.93E-02	↓EDN1, ↓ERBB2, ↓TRAF2
GSK690693	chemical drug				2.19E-02	↓ERBB2
Ampa Receptor	complex				2.19E-02	↓ERBB2
STC1	kinase				2.19E-02	↓POU1F1
BDKRB1	g-protein coupled receptor				2.19E-02	↓EDN1
LRIG1	other				2.19E-02	↓ERBB2
DNAJA3	other				2.19E-02	↓ERBB2
ERBB2IP	other				2.19E-02	↓ERBB2
MUSK	kinase				2.19E-02	↓ERBB2
SERPIND1	other				2.19E-02	↓KLF5
HLA-B	transmembrane receptor				2.19E-02	↓EDN1
tyrphostin AG825	chemical - kinase inhibitor				2.19E-02	↓ERBB2
canrenoate potassium	chemical drug				2.19E-02	↓EDN1

R5020	chemical reagent				2.33E-02	↓ERBB2, ↑FKBP5
SIRT1	transcription regulator				2.34E-02	↑MGAT2, ↓PER1, ↓TRAF2
NRG2	growth factor				2.39E-02	↓EDN1, ↓ERBB2
calcitriol	chemical drug		-1.000		2.50E-02	↓CHAF1A, ↓EDN1, ↓ERBB2, ↑NUP43, ↓POU1F1
Arnt-Hif1a	complex				2.55E-02	↓EDN1
REL/RELA/RELB	group				2.55E-02	↓TRAF2
WP1066	chemical drug				2.55E-02	↓EDN1
KDM3A	transcription regulator				2.55E-02	↓EDN1
enzalutamide	chemical drug				2.55E-02	↑FKBP5
miR-138-5p (miRNAs w/seed GCUGGUG)	mature microrna				2.55E-02	↓ROCK2
MLLT3	other				2.55E-02	↓EDN1
NPAS2	transcription regulator				2.55E-02	↓PER1
Elf5	other				2.55E-02	↓ERBB2
EWSR1	other				2.55E-02	↓ERBB2
diazepam	chemical drug				2.55E-02	↓PER1
PP2/AG1879 tyrosine kinase inhibitor	chemical - kinase inhibitor				2.75E-02	↓EDN1, ↓PER1
PLG	peptidase				2.89E-02	↓EDN1, ↓NOD1
FLT1	kinase				2.89E-02	↓APLP2, ↓TNIP1
novobiocin	chemical drug				2.91E-02	↓ERBB2
PROP1	transcription regulator				2.91E-02	↓POU1F1
FRS2	other				2.91E-02	↓ERBB2
SFN	other				2.91E-02	↓ERBB2
YBX3	transcription regulator				2.91E-02	↓ERBB2
CRTC1	transcription regulator				2.91E-02	↓PER1
PA2G4	transcription regulator				2.91E-02	↓ERBB2
diltiazem	chemical drug				2.91E-02	↓EDN1

cerulenin	chemical drug				2.91E-02	↓ERBB2
RHOA	enzyme				3.12E-02	↓ERBB2, ↓ROCK2
HTR7	g-protein coupled receptor				3.26E-02	↓STAB1
HTR2B	g-protein coupled receptor				3.26E-02	↓ERBB2
NEK7	kinase				3.26E-02	↓EDN1
LHX3	transcription regulator				3.26E-02	↓POU1F1
ERP29	transporter				3.26E-02	↓PARD3
TRAF1	other				3.26E-02	↓TRAF2
benazepril	chemical drug				3.26E-02	↓EDN1
farglitazar	chemical drug				3.26E-02	↓EDN1
betaine	chemical - endogenous mammalian				3.26E-02	↓MIOX
spironolactone	chemical drug				3.27E-02	↓EDN1, ↓POU1F1
beraprost	chemical drug				3.62E-02	↓EDN1
mGluR	group				3.62E-02	↓ERBB2
Atrial Natriuretic Peptide	group				3.62E-02	↓EDN1
BMS-754807	chemical drug				3.62E-02	↓ERBB2
MXD1	transcription regulator				3.62E-02	↓EDN1
ZFHX3	transcription regulator				3.62E-02	↓POU1F1
MIR320	group				3.62E-02	↓EDN1
RV 538	chemical reagent				3.62E-02	↓ATP13A2
levamisole	chemical drug				3.62E-02	↓EDN1
BQ-788	chemical drug				3.62E-02	↓EDN1
FN1	enzyme				3.62E-02	↓APLP2, ↓PPP6R2, ↓TNIP1
FOXP3	transcription regulator				3.92E-02	↓CHAF1A, ↓ERBB2
sorbitol	chemical - endogenous mammalian				3.97E-02	↓MIOX
WHSC1	enzyme				3.97E-02	↓BACE2

hemoglobin	complex				3.97E-02	↓EDN1
WDR5	other				3.97E-02	↓ERBB2
HIF3A	transcription regulator				3.97E-02	↓EDN1
XRCC5	enzyme				3.97E-02	↑FKBP5
ELK3	transcription regulator				3.97E-02	↓ERBB2
toremifene	chemical drug				3.97E-02	↓ERBB2
catecholamine	chemical - other				3.97E-02	↓ERBB2
baclofen	chemical drug				3.97E-02	↓PER1
KT5823	chemical - kinase inhibitor				3.97E-02	↓EDN1
Pkc(s)	group				4.00E-02	↓EDN1, ↓PER1, ↓POU1F1
HIST1H1T	other				4.00E-02	↓PCCB, ↓SMARCE1
Hist1h1a	other				4.00E-02	↓PCCB, ↓SMARCE1
cycloheximide	chemical reagent				4.17E-02	↓EDN1, ↓KLF5, ↓POU1F1, ↓TNIP1
triamcinolone acetonide	chemical drug				4.25E-02	↓DGKE, ↑FKBP5, ↓PER1
LMNA	other				4.25E-02	↓EDN1, ↓EFNB3, ↓STAB1
actinomycin D	chemical drug				4.25E-02	↓EDN1, ↓POU1F1, ↓ROCK2
PARP1	enzyme				4.26E-02	↓ERBB2, ↑FKBP5
GATA5	transcription regulator				4.33E-02	↓EDN1
metoprolol	chemical drug				4.33E-02	↓EDN1
androstenediol	chemical - endogenous mammalian				4.33E-02	↑FKBP5
dihydrotestosterone	chemical - endogenous mammalian		0.372	bias	4.34E-02	↓ATP13A2, ↓ERBB2, ↑FKBP5, ↓PER1, ↓SLC7A8
NRG1	growth factor				4.35E-02	↓EDN1, ↓ERBB2, ↓PCCB
NKX2-3	transcription regulator				4.45E-02	↓EDN1, ↓FAM172A, ↓SHMT2
tetrachlorodibenzodioxin	chemical toxicant				4.54E-02	↓EDN1, ↓EPS15L1, ↓ERBB2, ↓PRCC
CREB1	transcription		-1.067		4.55E-02	↓CGREF1, ↓EDN1, ↓GABRA5,

	regulator					↓KLF5, ↓PER1
alvespimycin	chemical drug				4.68E-02	↓ERBB2
COL1A1	other				4.68E-02	↓EDN1
XRCC6	enzyme				4.68E-02	↑FKBP5
PER2	transcription regulator				4.68E-02	↓PER1
LPAR1	g-protein coupled receptor				4.68E-02	↓KLF5
CRY2	enzyme				4.68E-02	↓PER1
CRY1	enzyme				4.68E-02	↓PER1
clasto-lactacystin beta-lactone	chemical - protease inhibitor				4.68E-02	↓NR1I2
hyperforin	chemical drug				4.68E-02	↓NR1I2
naltrindole	chemical drug				4.68E-02	↓CDK16
L-glutamic acid	chemical - endogenous mammalian				4.79E-02	↓PER1, ↓ROCK2
CDX2	transcription regulator				4.97E-02	↓KLF5, ↓SLC7A8

Supplementary Table 12 Primer sequences, annealing temperatures and product sizes for PCR reactions.

Gene Symbol	Accession number	Primer sequence		Annealing Temperature	Product length
		Forward	Reverse		
mtDNA	AJ002189	CTC AAC CCT AGC AGA AAC CA	TTA GTT GGT CGT ATC GGA ATC	55	254
ACT-B	XM_003124280.3	GTGGACATCAGGAAGGACCTCTA	ATGATCTTGATCTTCATGGTGCT	60	130
OCT4 (POU5F1)	NM_001113060	CACCTCAGGTCGGAGTGG	AGCTTGGCAAATTGTTCGA	58	226
SOX2	NM_001123197.1	AACCAGAAGAACAGCCCAGA	CGGGGCCGGTATTTATAATC	58	246
REX1	XM_003359865	TTTCTGAGTACGTGCCAGGCAA	GAACGGAGAGATGCTTTCTCAGAG	62	201
Nanog	NM_001129971.1	AGGGCTCAGCCAGTACAGAA	TGATTTTCCCCAGCAGTTTC	60	316
CDX2	NM_001278769.1	CTCGGCAGCCAAGTGAAA	TCCTCTCCTTCGCTCTGC	62	198
ND1	NC_000845.1	CGGACCTTTCGCCATATT	GTATCGGAATCGTGGGTATG	60	195
ATP6	NC_000845.1	CACCCACCACACAACTATC	CTAGGGCTACTGGTTGAATAAA	60	191
TFAM	NM_001130211.1	CCT TTC CAC ATA CAA CCA TCGA	TCC AGA ACT CAT CTT GGT AAA TTC C	62	80

Supplementary Figures

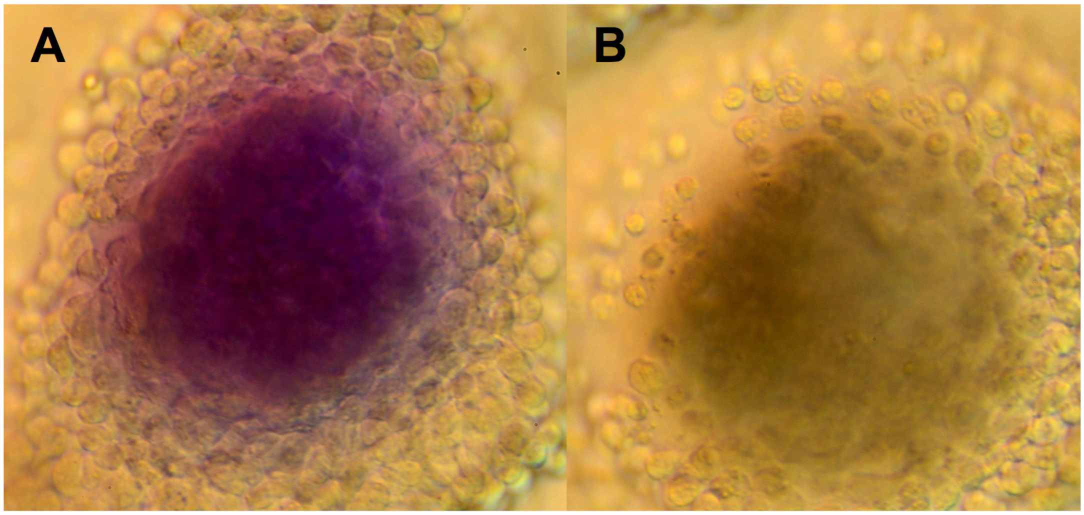

Supplementary Figure 1 Brilliant Cresyl Blue (BCB) staining of cumulus-oocyte complexes before IVM. BCB dye is retained in BCB^+ oocytes (**a**) and reduced to a colourless compound in BCB^- oocytes (**b**).

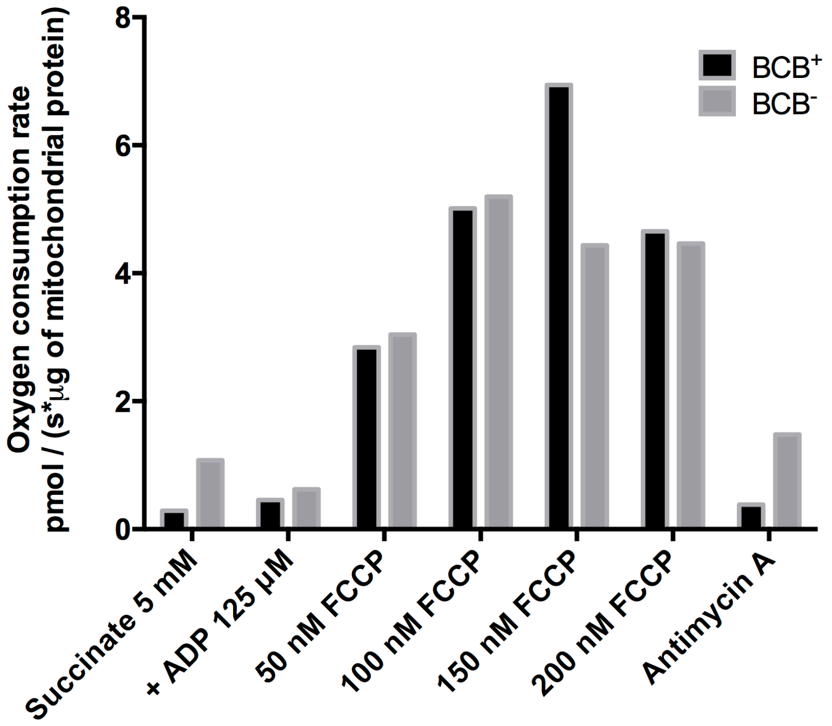

Supplementary Figure 2 O_2 consumption rates for mitochondrial extracts isolated from BCB^+ and BCB^- oocytes after 44hr of IVM. Mitochondrial extracts were subjected to 5 mM succinate, followed by ADP and increased FCCP concentrations (50 mM increments). Respiration was abolished by addition of Antimycin A.

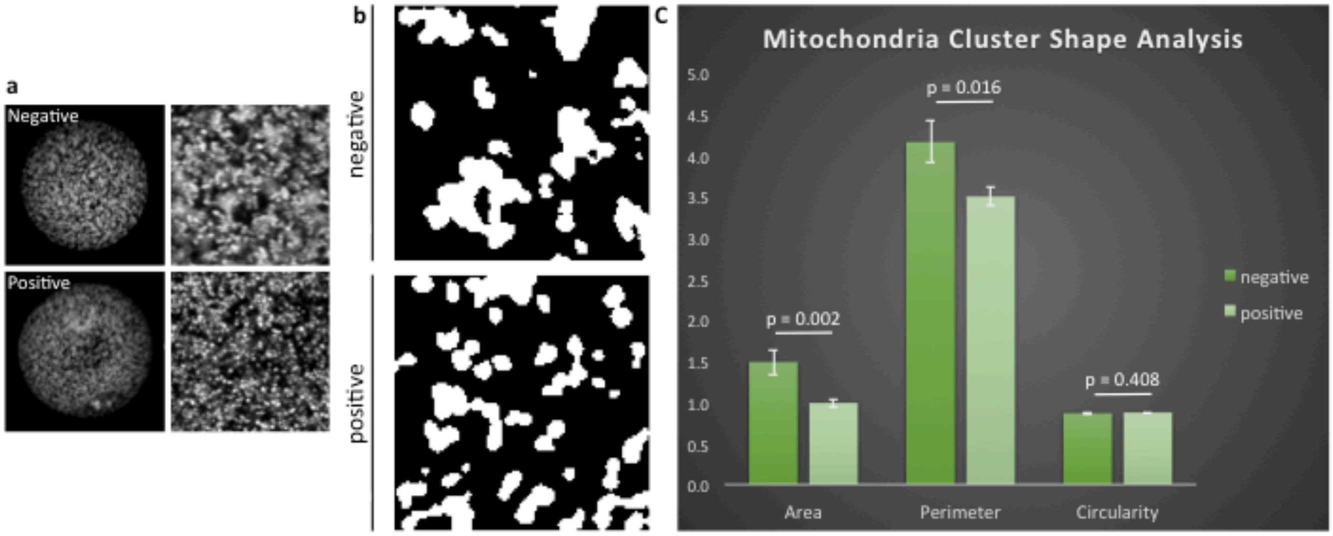

Supplementary Figure 3 Shape analysis of mitochondrial clusters. a) BCB^- and BCB^+ MII oocytes are stained with MitoTracker Red. b) Region of interests (ROIs) were thresholded and binarized. Cluster area, perimeter and circularity were chosen as parameters for characterization. c) Cluster analysis was performed using two-tailed t-test.

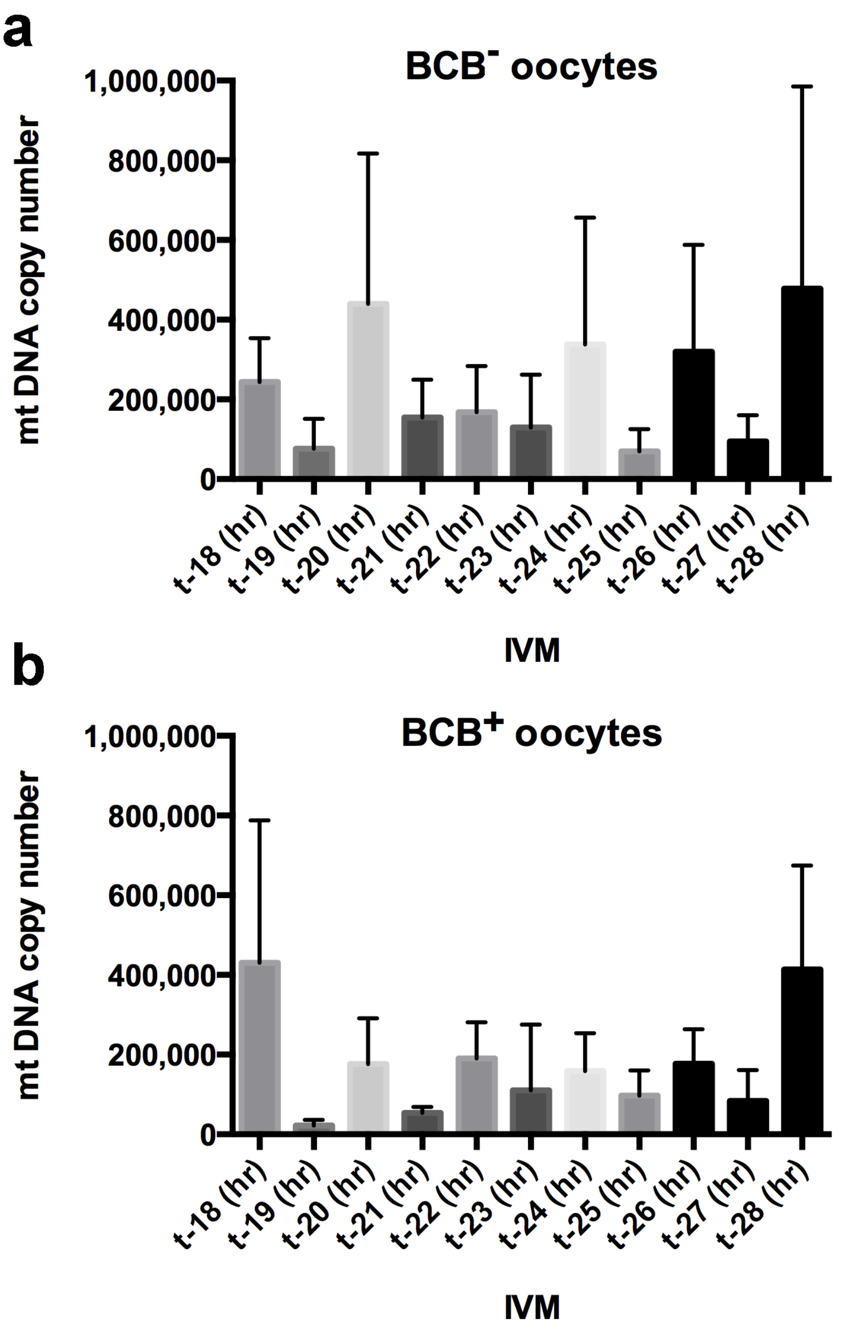

Supplementary Figure 4 Mean (± SEM) of mtDNA copy number for BCB^+ (**a**) and BCB^- (**b**) oocytes. Oocytes were collected every hour between 18h and 28h of IVM (n = 5-10 oocytes per time point).

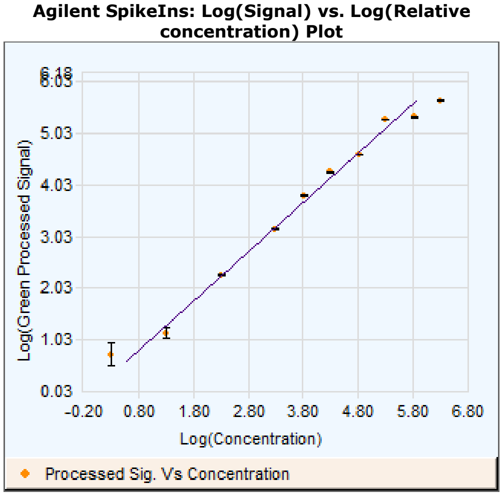

Agilent Spike-In Concentration-Response Statistics Linear Range Statistics	
Low Signal	0.61
High Signal	5.68
Low Relative Concentration	0.59
High Relative Concentration	5.89
Slope	0.96
R^2 Value	0.99

Signal Detection Limit Statistics	
Saturation Point	5.89
Low Threshold	0.31
Low Threshold Error	0.24
Spike-In Detection Limit	1.04

Supplementary Figure 5 Correlation between exogenous spike-in RNA concentrations added to the sample and detected by microarray. The microarray is designed with probes to detect different RNA spike-ins that are added at known concentrations (Concentration) to each sample before RNA extraction. After sample and spike-in RNA extraction, total RNA was amplified to produce sufficient amounts for labelling (green) and hybridization on the microarray (Green process signal). A high correlation score indicates low technical bias in RNA processing.

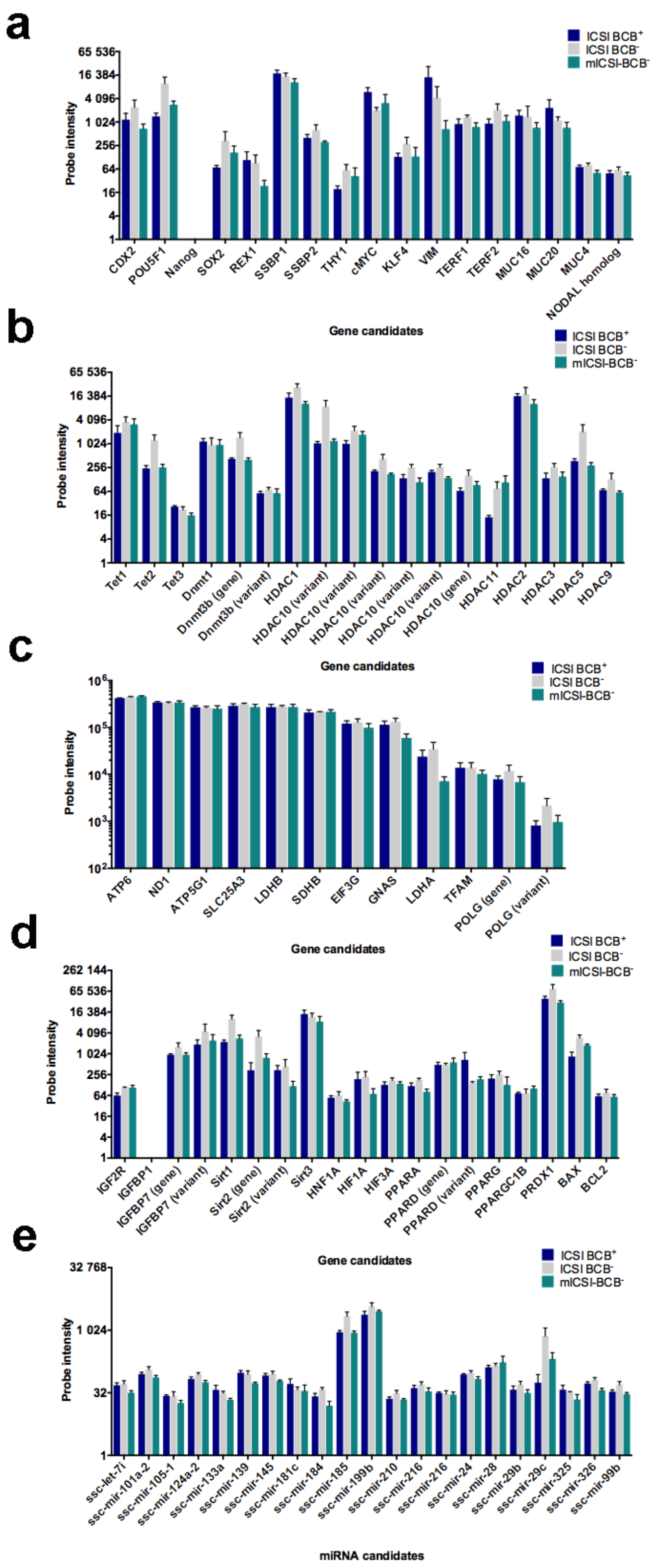

a
ICSI BCB+
ICSI BCB-
mICSI-BCB-
Probe intensity
65 536
16 384
4 096
1 024
256
64
16
4
1
CDX2
POU5F1
Nanog
SOX2
REX1
SSBP1
SSBP2
THY1
cMYC
KLF4
VIM
TERF1
TERF2
MUC16
MUC20
MUC4
NODAL homolog
Gene candidates
b
ICSI BCB+
ICSI BCB-
mICSI-BCB-
Probe intensity
65 536
16 384
4 096
1 024
256
64
16
4
1
Tet1
Tet2
Tet3
Dnmt1
Dnmt3b (gene)
Dnmt3b (variant)
HDAC1
HDAC10 (variant)
HDAC10 (variant)
HDAC10 (variant)
HDAC10 (variant)
HDAC10 (variant)
HDAC10 (gene)
HDAC11
HDAC2
HDAC3
HDAC5
HDAC9
Gene candidates
c
ICSI BCB+
ICSI BCB-
mICSI-BCB-
Probe intensity
10^6
10^5
10^4
10^3
10^2
ATP6
ND1
ATP5G1
SLC25A3
LDHB
SDHB
EIF3G
GNAS
LDHA
TFAM
POLG (gene)
POLG (variant)
Gene candidates
d
ICSI BCB+
ICSI BCB-
mICSI-BCB-
Probe intensity
262 144
65 536
16 384
4 096
1 024
256
64
16
4
1
IGF2R
IGFBP1
IGFBP7 (gene)
IGFBP7 (variant)
Sirt1
Sirt2 (gene)
Sirt2 (variant)
Sirt3
HNF1A
HIF1A
HIF3A
PPARA
PPARD (gene)
PPARD (variant)
PPARG
PPARGC1B
PRDX1
BAX
BCL2
Gene candidates
e
ICSI BCB+
ICSI BCB-
mICSI-BCB-
Probe intensity
32 768
1 024
32
1
ssc-let-7i
ssc-mir-101a-2
ssc-mir-105-1
ssc-mir-124a-2
ssc-mir-133a
ssc-mir-139
ssc-mir-145
ssc-mir-181c
ssc-mir-184
ssc-mir-185
ssc-mir-199b
ssc-mir-210
ssc-mir-216
ssc-mir-216
ssc-mir-24
ssc-mir-28
ssc-mir-29b
ssc-mir-29c
ssc-mir-325
ssc-mir-326
ssc-mir-99b
miRNA candidates

Supplementary Figure 6 Normalized microarray intensity values for genes associated with blastocyst development for ICSI BCB^{+}, ICSI BCB^{-} and mICSI BCB^{+} derived blastocysts. **a**) pluripotency; **b**) epigenetic reprogramming; **c** and **d**) energy metabolism; and **e**) microRNA genes.

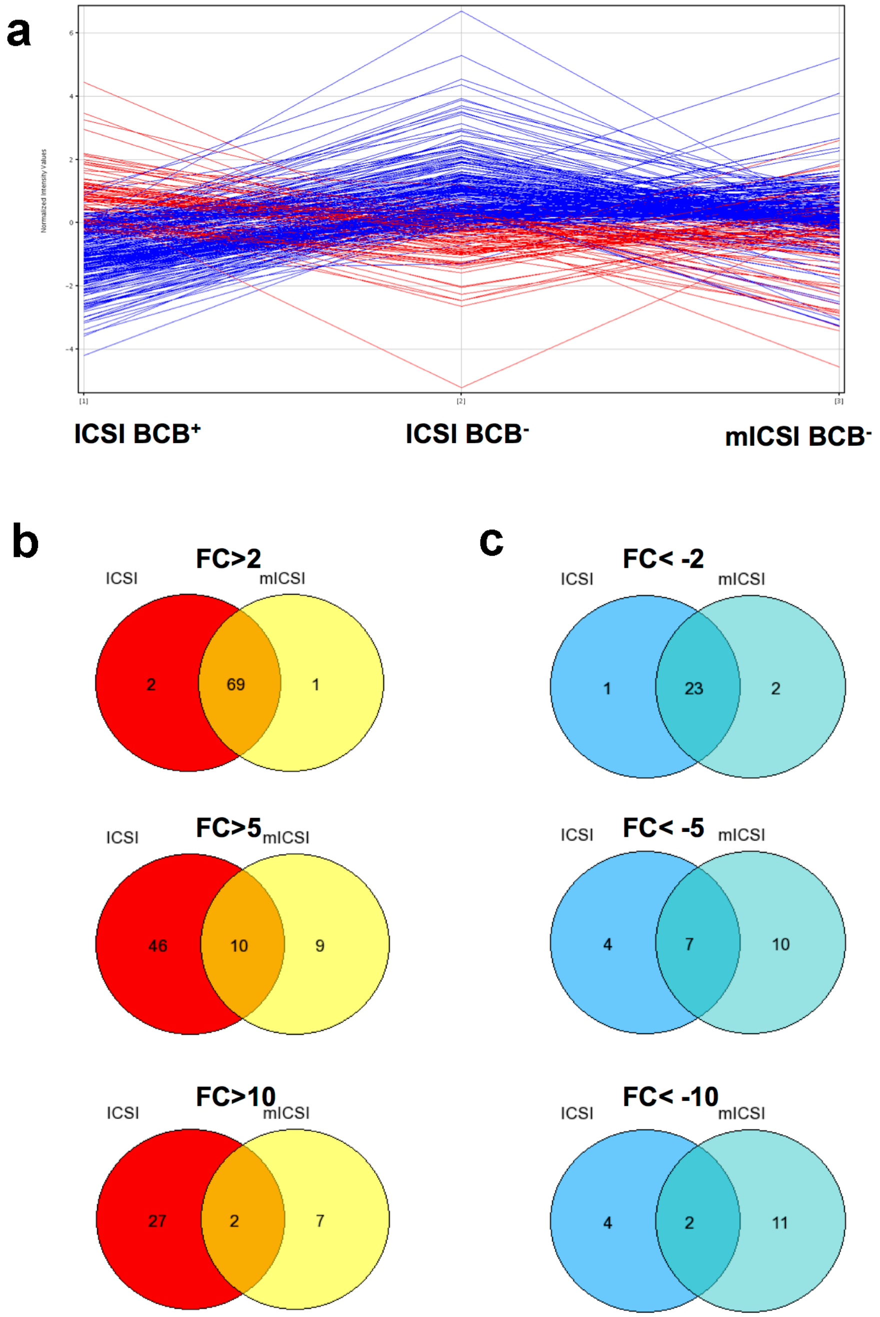

Supplementary Figure 7 Normalized fold-change profiles for differentially expressed genes for ICSI BCB$^+$, ICSI BCB$^-$ and mICSI BCB$^-$ derived blastocysts. (**a**) Analysis was conducted using ANOVA, FC>2 (abs) p<0.01. (**b**) Number of up-regulated and (**c**) down-regulated genes in ICSI BCB$^-$ and mICSI BCB$^-$ blastocysts at p<0.01 (FC relative to BCB$^+$ ICSI).

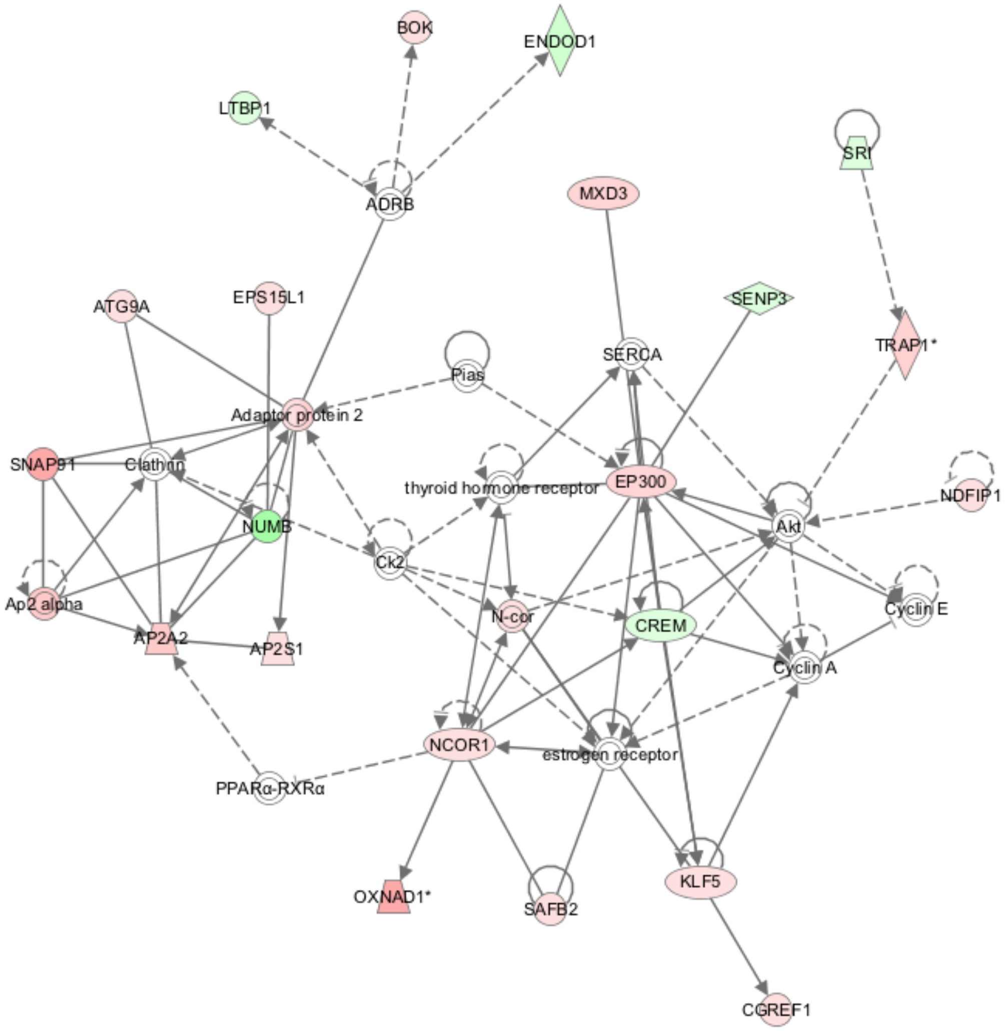

Supplementary Figure 8 Differentially expressed genes between ICSI BCB$^-$ and ICSI BCB$^+$ blastocysts affecting the cellular assembly and organisation network. A comparison of differentially expressed genes between ICSI BCB$^-$ and ICSI BCB$^+$ blastocysts demonstrated the up (red) and down (green) regulation of gene expression for ICSI BCB$^-$ blastocysts.

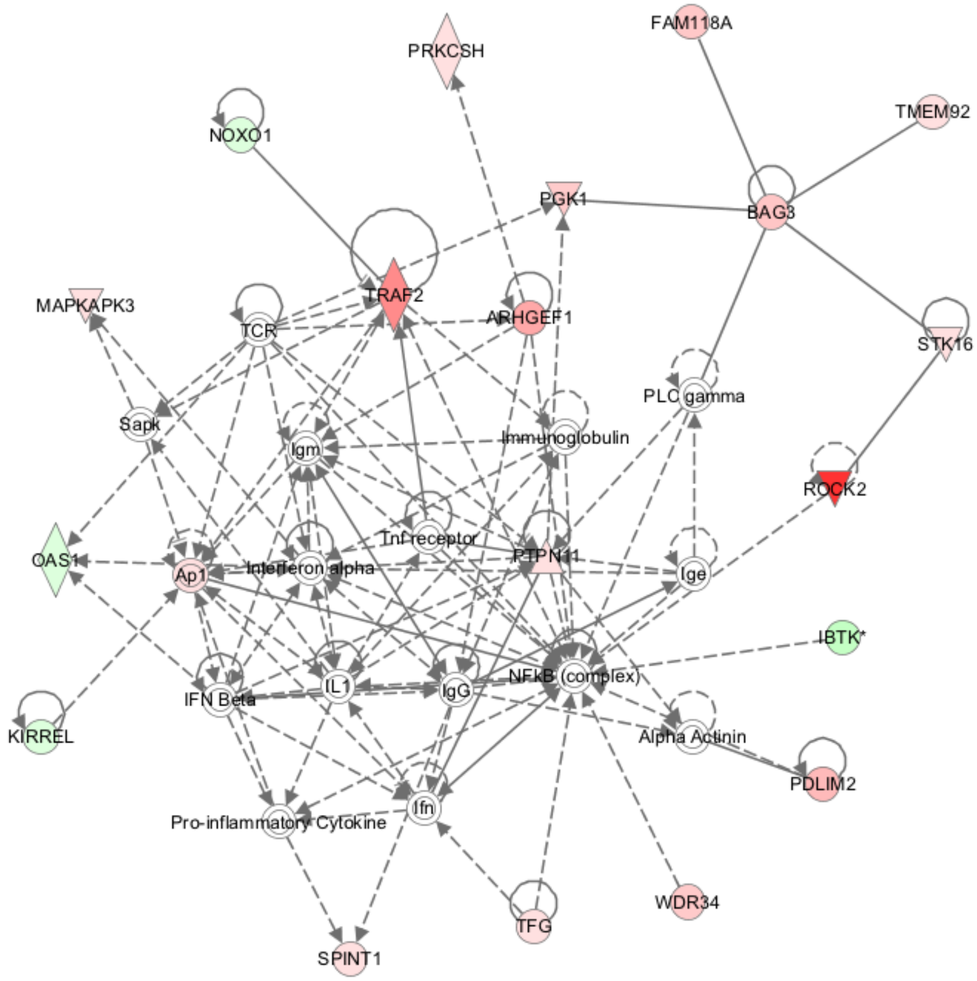

Supplementary Figure 9 Differentially expressed genes between ICSI BCB$^-$ and ICSI BCB$^+$ blastocysts affecting the cell morphology network. A comparison of differentially expressed genes between ICSI BCB$^-$ and ICSI BCB$^+$ blastocysts demonstrated the up (red) and down (green) regulation of gene expression for ICSI BCB$^-$ blastocysts.

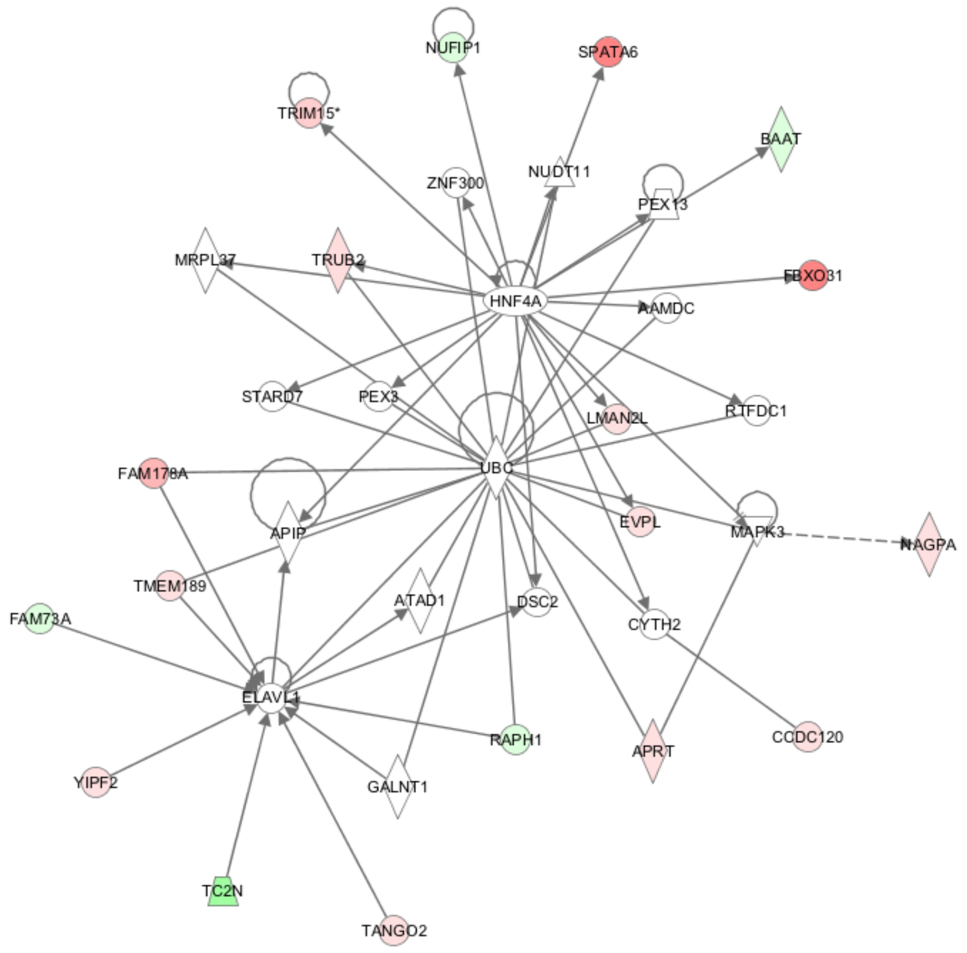

Supplementary Figure 10 Differentially expressed genes between ICSI BCB$^-$ and ICSI BCB$^+$ blastocysts affecting the amino acid metabolism network. A comparison of differentially expressed genes between ICSI BCB$^-$ and ICSI BCB$^+$ blastocysts demonstrated the up (red) and down (green) regulation of gene expression for ICSI BCB$^-$ blastocysts.

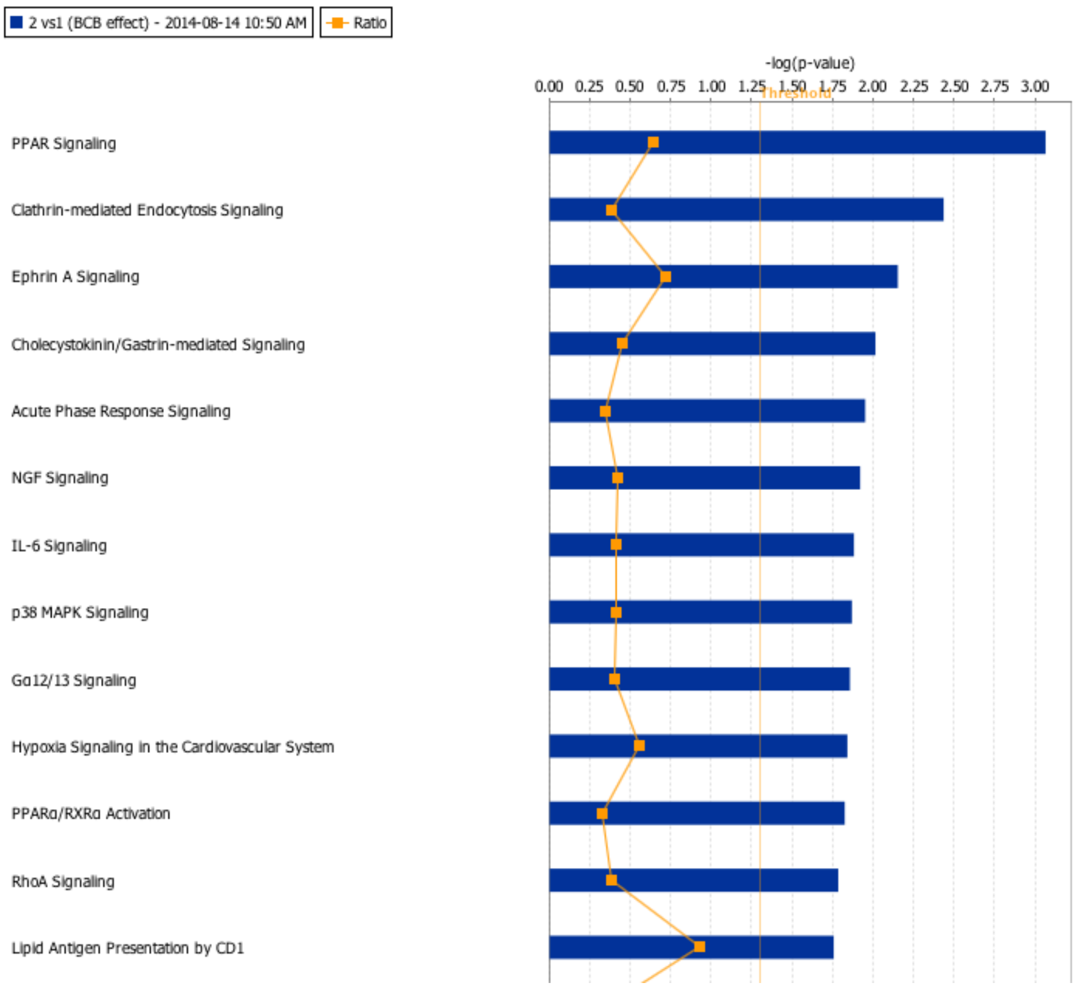

Supplementary Figure 11 Canonical pathways significantly enriched in differentially expressed genes between ICSI BCB$^-$ and ICSI BCB$^+$ blastocysts.

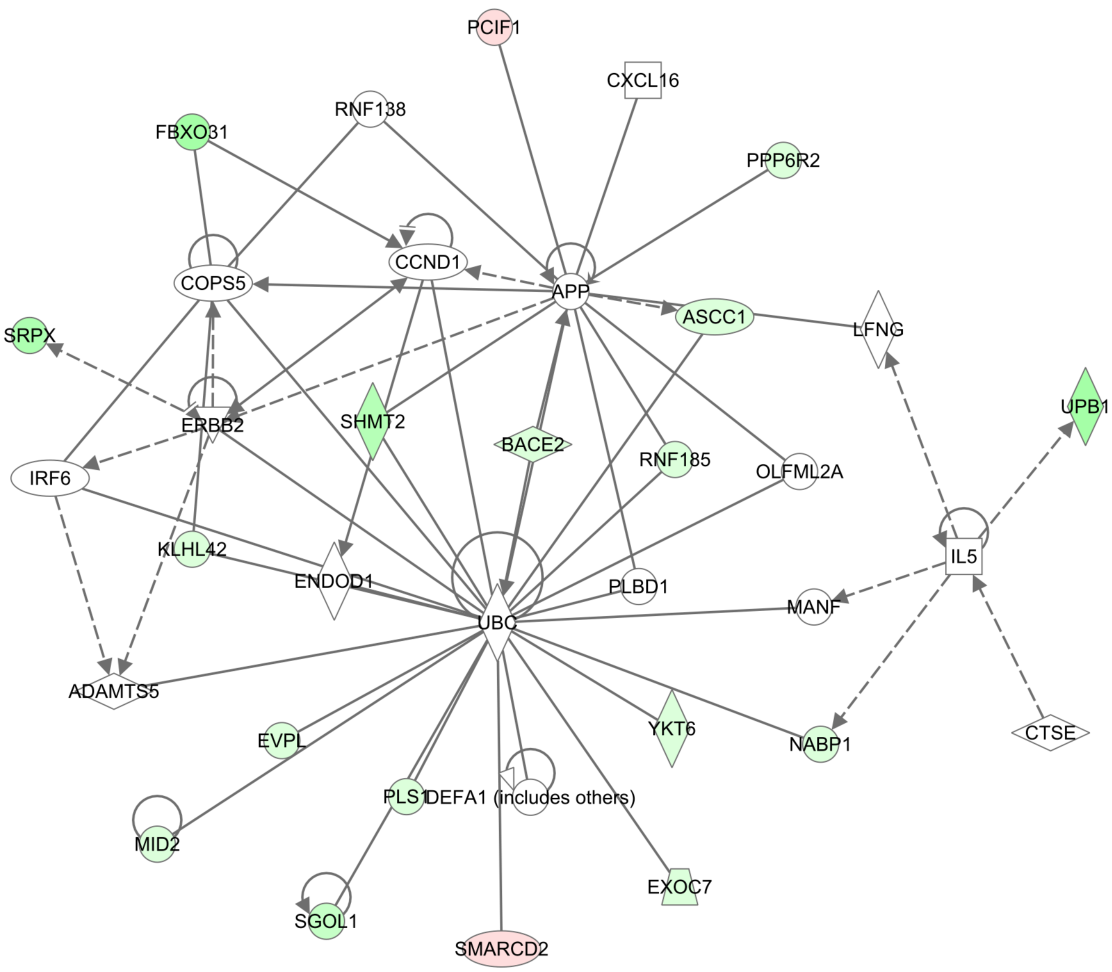

Supplementary Figure 12 Differentially expressed genes between mICSI BCB$^-$ and ICSI BCB$^-$ blastocysts affecting the cellular movement network. A comparison of differentially expressed genes between mICSI BCB$^-$ and ICSI BCB$^-$ blastocysts demonstrated the up (red) and down (green) regulation of genes.

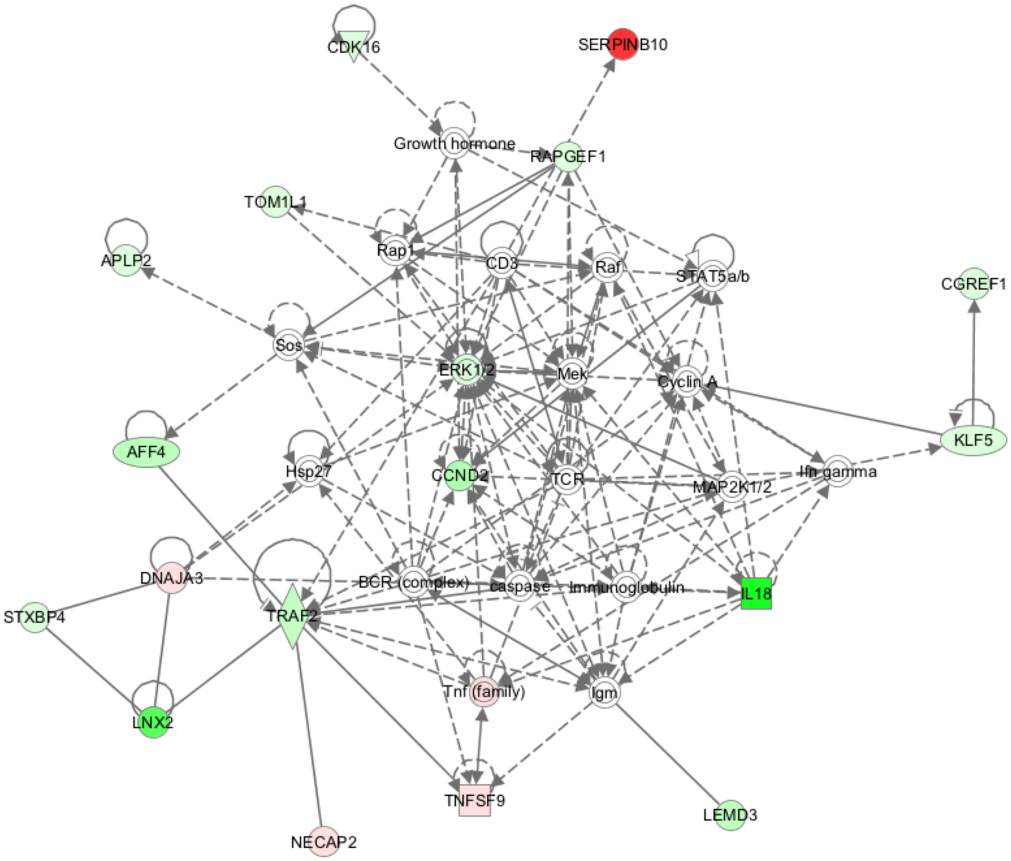

Supplementary Figure 13 Differentially expressed genes between mICSI BCB$^-$ and ICSI BCB$^-$ blastocysts affecting the cellular development network. A comparison of differentially expressed genes between mICSI BCB$^-$ and ICSI BCB$^-$ blastocysts demonstrated the up (red) and down (green) regulation of genes.

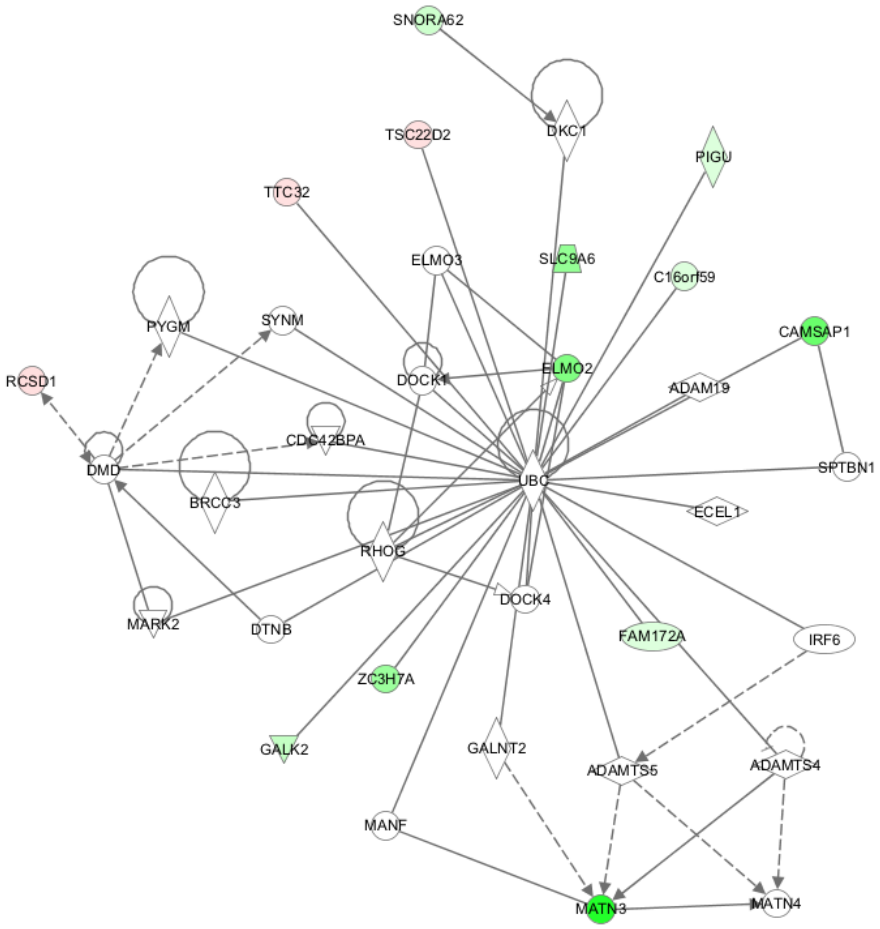

Supplementary Figure 14 Differentially expressed genes between mICSI BCB$^-$ and ICSI BCB$^-$ blastocysts affecting the cellular morphology network. A comparison of differentially expressed genes between mICSI BCB$^-$ and ICSI BCB$^-$ blastocysts demonstrated the up (red) and down (green) regulation of genes.

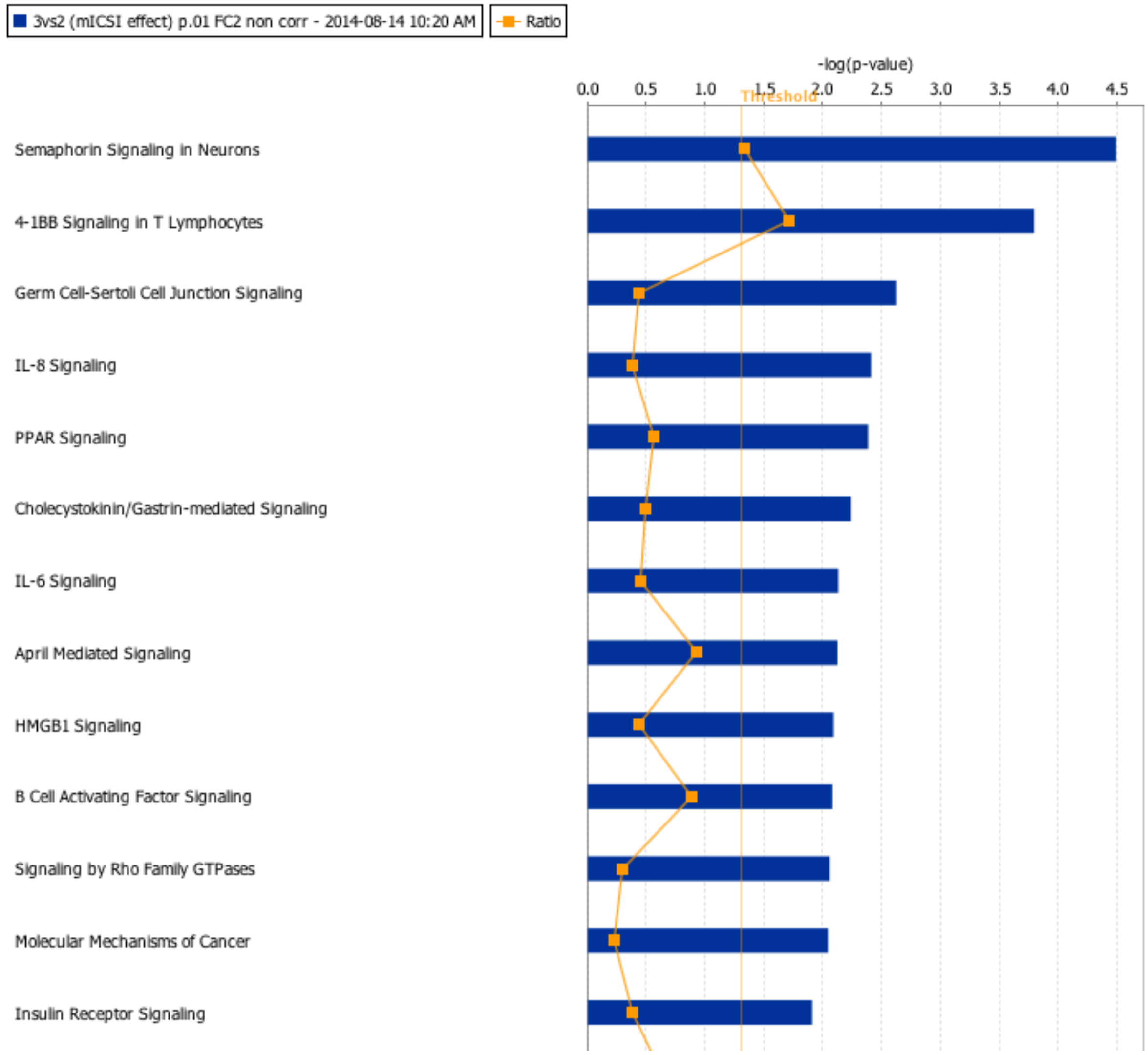

Supplementary Figure 15 Canonical pathways significantly enriched in differentially expressed genes between mICSI BCB$^-$ and ICSI BCB$^-$ blastocysts.

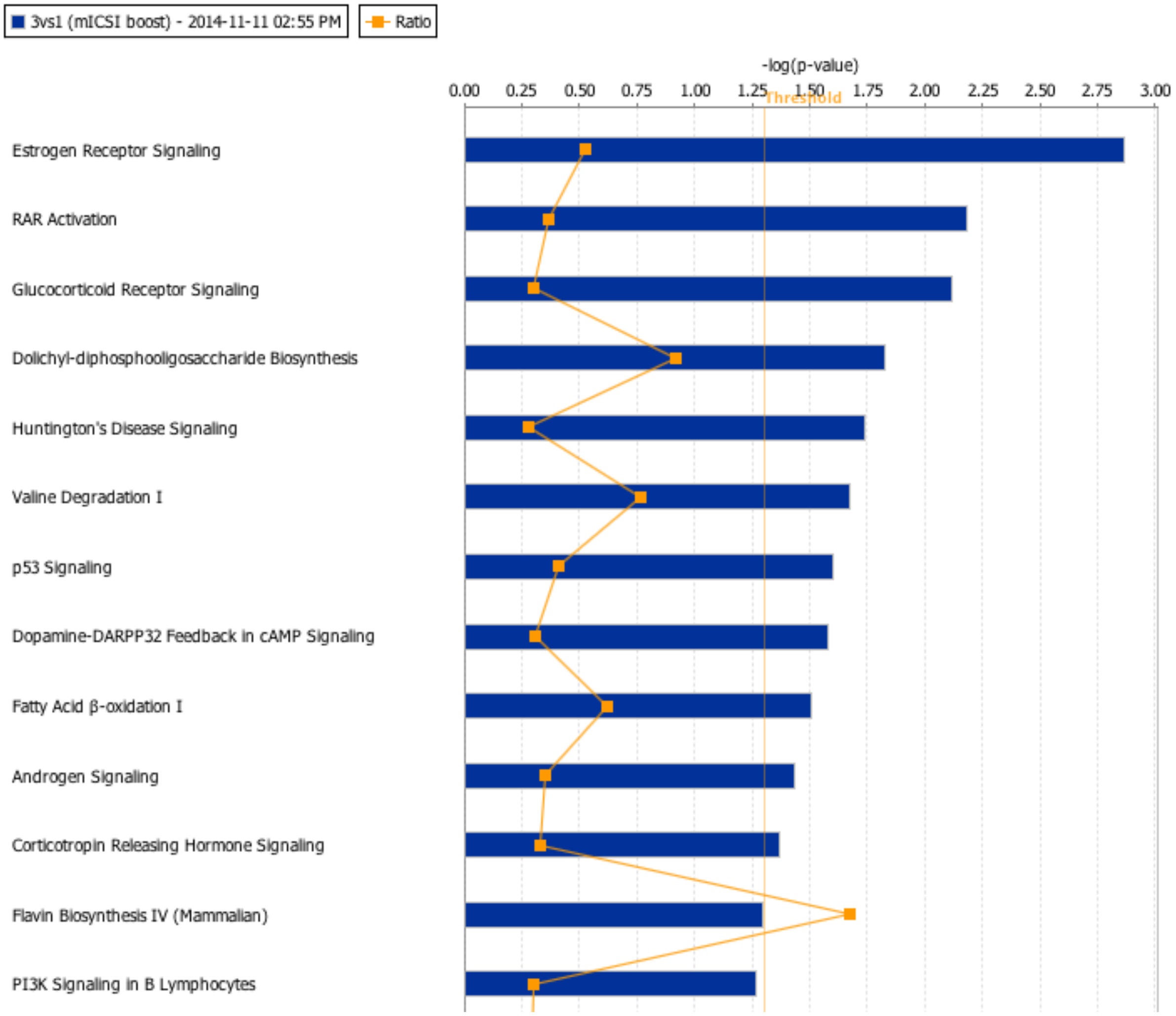

Supplementary Figure 16 Canonical pathways significantly enriched in differentially expressed genes between mICSI BCB$^-$ and ICSI BCB$^+$ blastocysts.

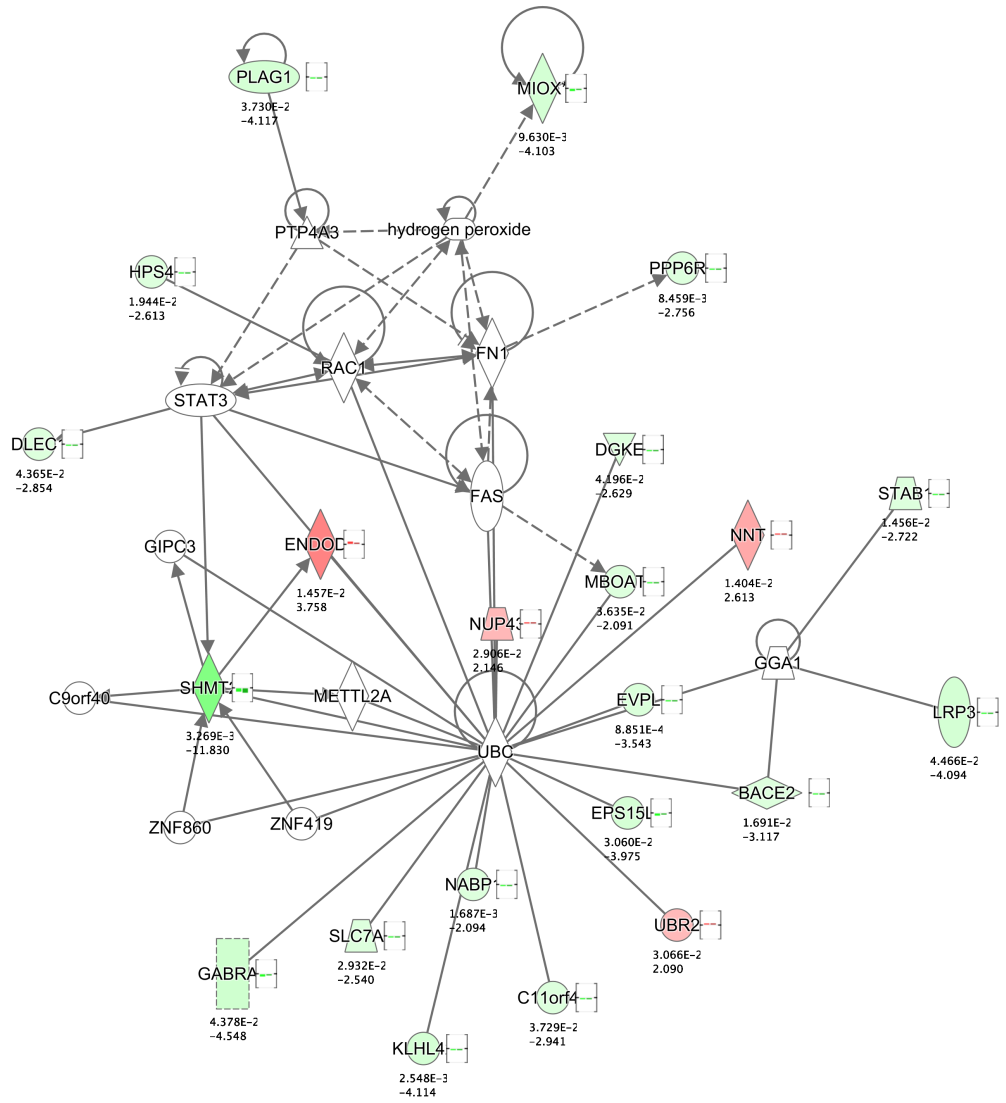

Supplementary Figure 17 Commonly expressed genes between ICSI-BCB$^+$ and mICSI-BCB$^-$ blastocysts but different to ICSI-BCB$^-$ blastocysts affecting the cell morphology network. The comparisons between ICSI-BCB$^+$ and ICSI-BCB$^-$, and mICSI-BCB$^-$ and ICSI-BCB$^-$ are shown by up (red) and down (green) regulation of genes. The comparisons between the two networks are overlaid to show differences in gene expression, which are indicated in the [ ].

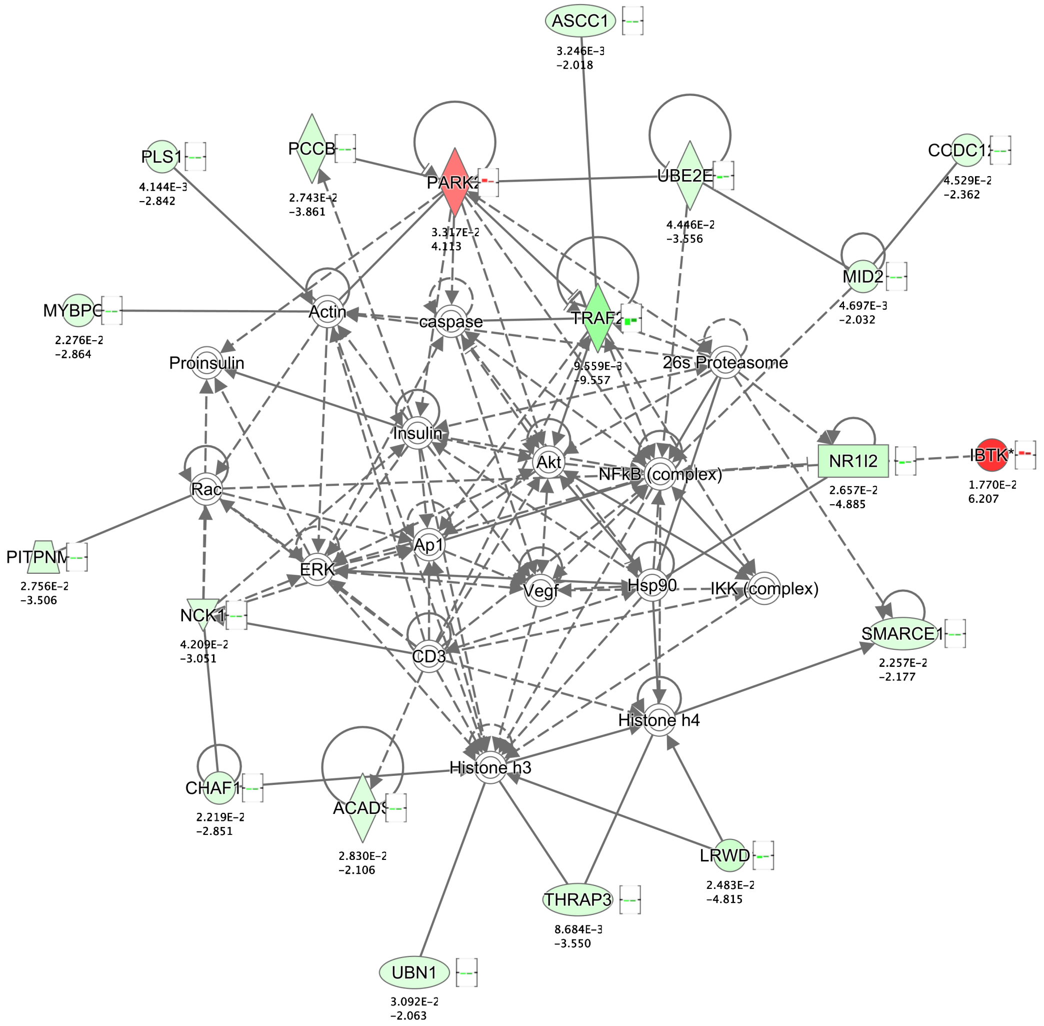

Supplementary Figure 18 Genes commonly expressed between ICSI-BCB^{+} and mICSI-BCB^{-} but different to ICSI-BCB^{-} blastocysts affecting the gene expression and protein synthesis network. The comparisons between ICSI-BCB^{+} and ICSI-BCB^{-} blastocysts and the mICSI-BCB^{-} and ICSI-BCB^{-} blastocysts are shown by the up (red) and down (green) regulation of genes. The comparisons of the two networks are overlaid to show differences in gene expression, which are indicated in the [ ].

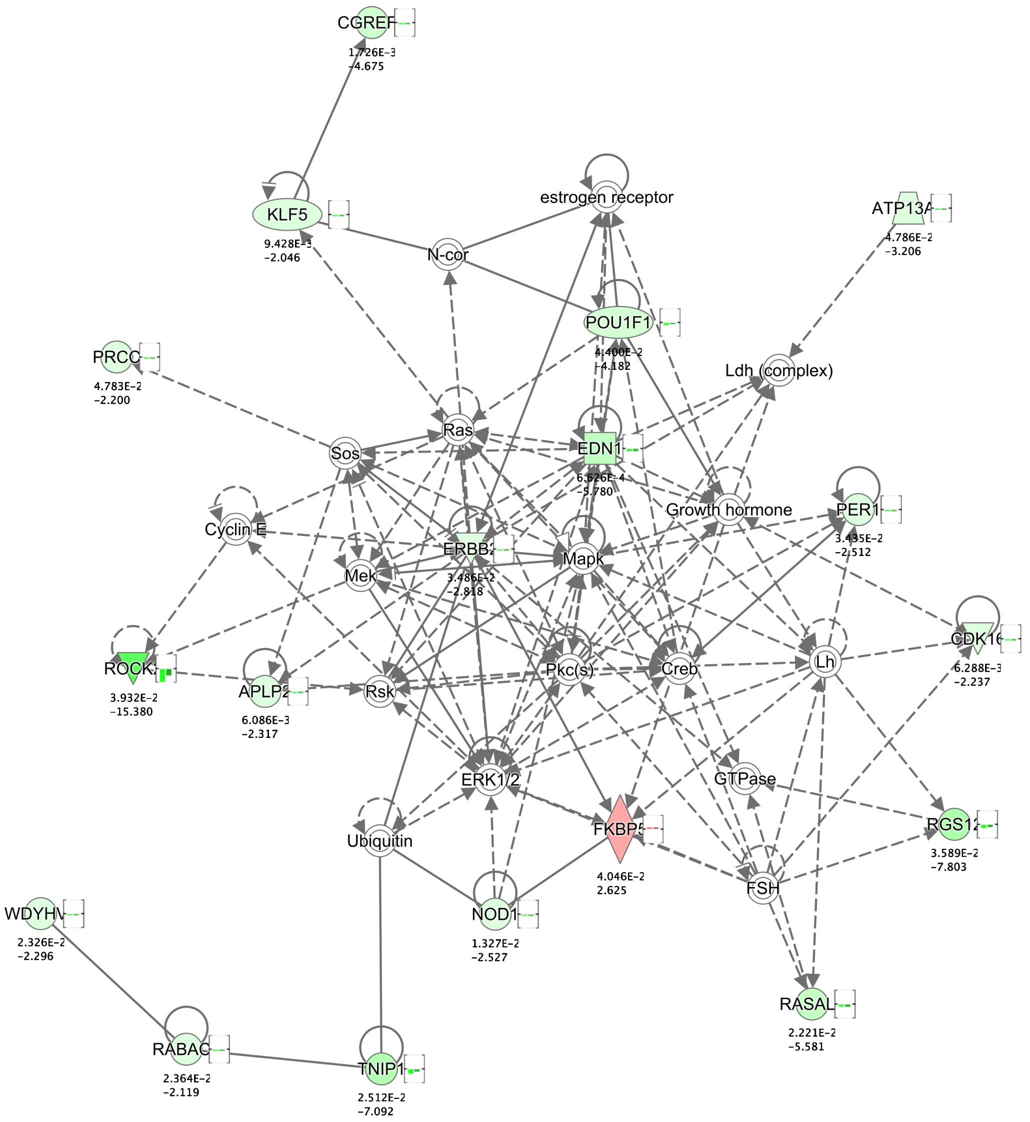

Supplementary Figure 19 Genes which are commonly expressed between ICSI-BCB$^+$ and mICSI-BCB$^-$, but different to ICSI-BCB$^-$ blastocysts affecting the cellular assembly and organization network. Although the comparisons between ICSI-BCB$^+$ and ICSI-BCB$^-$, as well as mICSI-BCB$^-$ and ICSI-BCB$^-$ were very similar, shown by the up (red) and down (green) regulation of genes. The comparisons of the two network are overlaid to show differences in gene expression, which are indicated in the [ ].

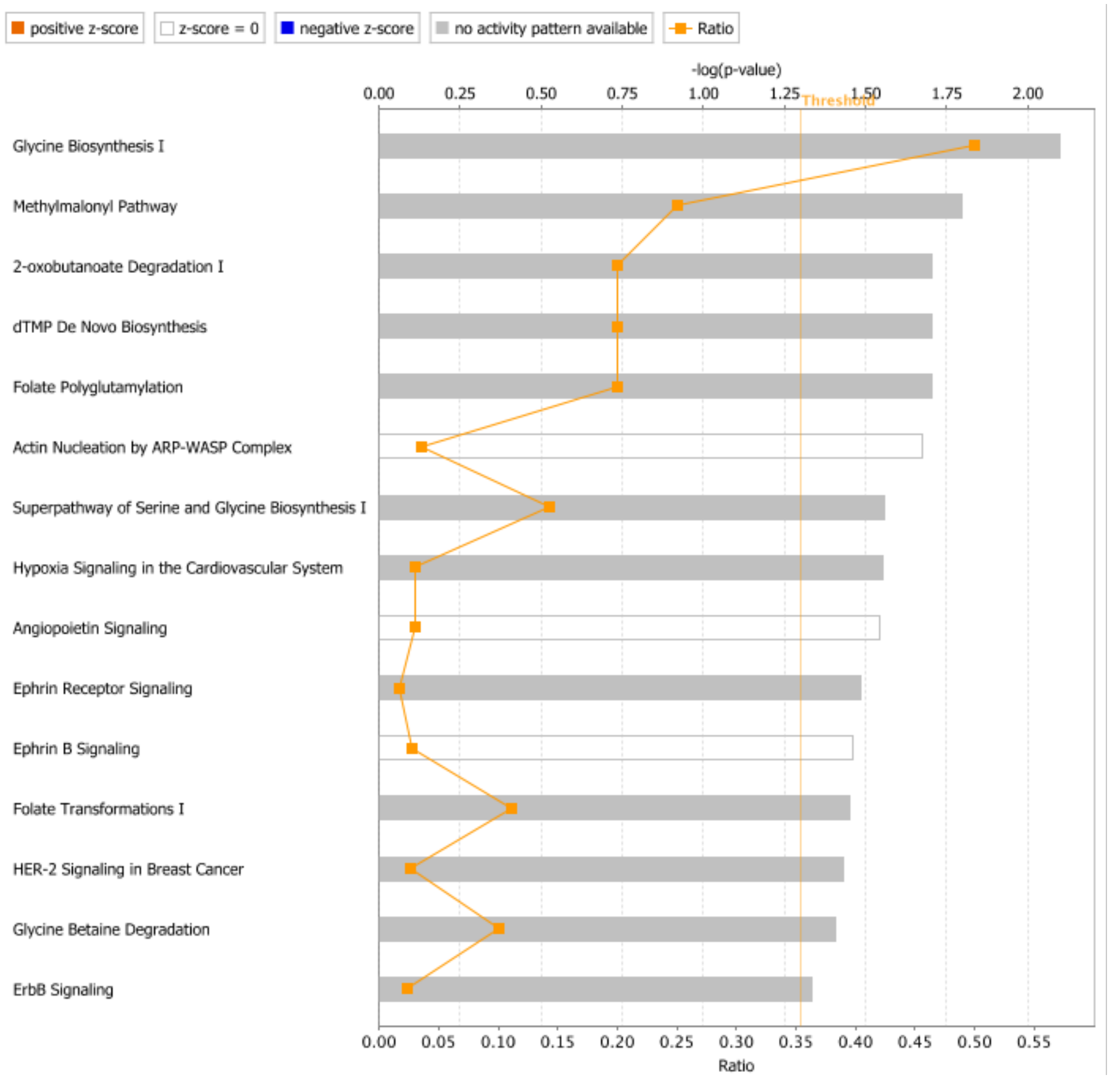

Supplementary Figure 20 Canonical pathways significantly enriched in commonly expressed genes between ICSI BCB^{+} and mICSI BCB^{-} but different to ICSI BCB^{-} blastocysts.

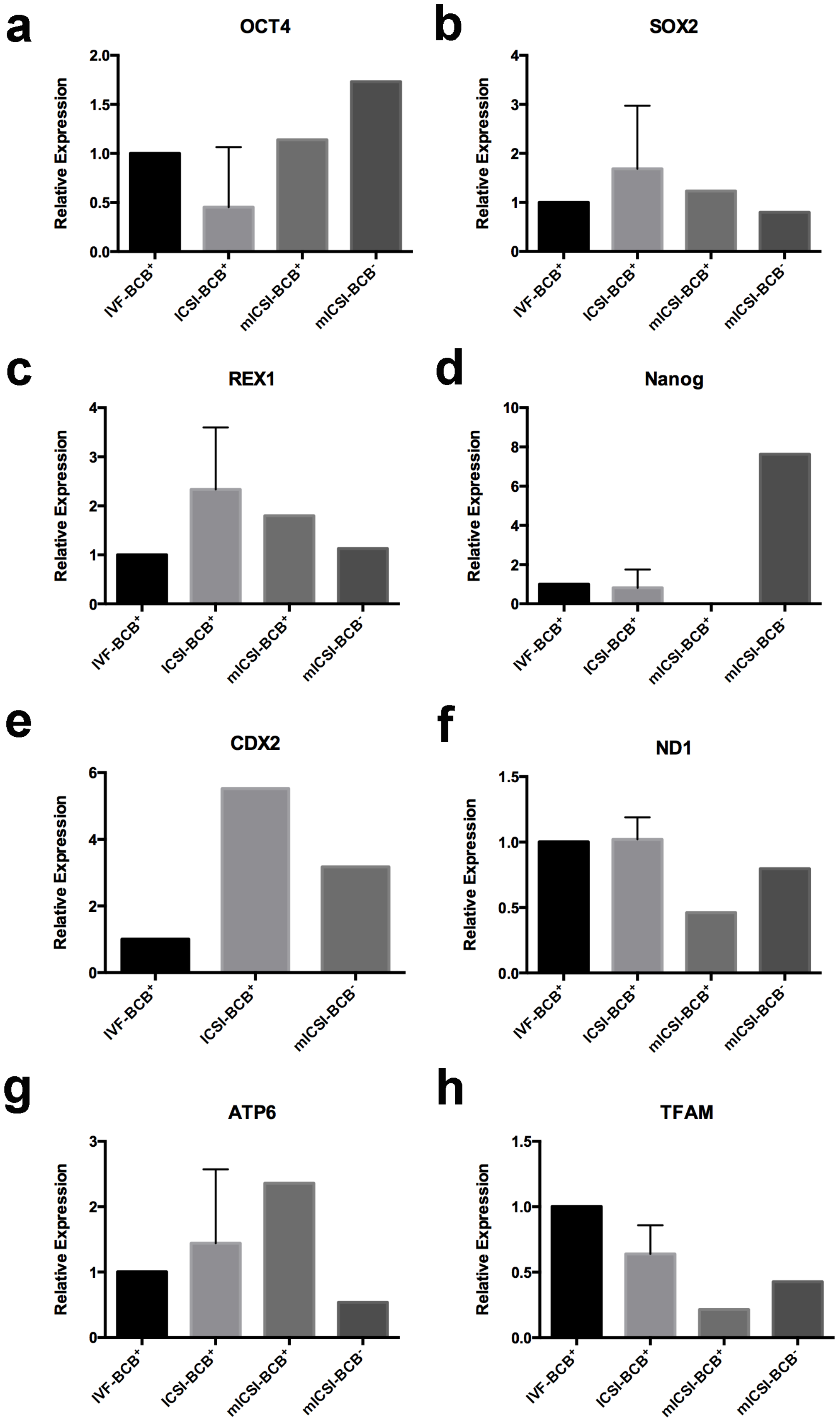

a
OCT4
Relative Expression
2.0
1.5
1.0
0.5
0.0
IVF-BCB+
ICSI-BCB+
mICSI-BCB+
mICSI-BCB-
b
SOX2
Relative Expression
4
3
2
1
0
IVF-BCB+
ICSI-BCB+
mICSI-BCB+
mICSI-BCB-
c
REX1
Relative Expression
4
3
2
1
0
IVF-BCB+
ICSI-BCB+
mICSI-BCB+
mICSI-BCB-
d
Nanog
Relative Expression
10
8
6
4
2
0
IVF-BCB+
ICSI-BCB+
mICSI-BCB+
mICSI-BCB-
e
CDX2
Relative Expression
6
4
2
0
IVF-BCB+
ICSI-BCB+
mICSI-BCB-
f
ND1
Relative Expression
1.5
1.0
0.5
0.0
IVF-BCB+
ICSI-BCB+
mICSI-BCB+
mICSI-BCB-
g
ATP6
Relative Expression
3
2
1
0
IVF-BCB+
ICSI-BCB+
mICSI-BCB+
mICSI-BCB-
h
TFAM
Relative Expression
1.5
1.0
0.5
0.0
IVF-BCB+
ICSI-BCB+
mICSI-BCB+
mICSI-BCB-

Supplementary Figure 21 Relative gene expression for early regulators of development in blastocysts derived from IVF, ICSI and mICSI. Real-time RT-PCR values were normalized to ACT-B values and compared to IVF-blastocysts as the reference point. IVF and ICSI-BCB$^+$ values represent the mean (± SEM) of gene expression levels from 2 pools of 5 blastocysts each. Values for the mICSI-BCB$^-$ group represent gene expression from one pool of 5 blastocysts each.
